# The Swi5–Sfr1 complex regulates Dmc1- and Rad51-driven DNA strand exchange proceeding through two distinct three-stranded intermediates by different mechanisms

**DOI:** 10.1093/nar/gkae841

**Published:** 2024-09-28

**Authors:** Kentaro Ito, Takahisa Maki, Shuji Kanamaru, Masayuki Takahashi, Hiroshi Iwasaki

**Affiliations:** Institute of Innovative Research, Tokyo Institute of Technology, Yokohama, Kanagawa 226-8503, Japan; Institute of Innovative Research, Tokyo Institute of Technology, Yokohama, Kanagawa 226-8503, Japan; Department of Life Science and Technology, School of Life Science and Technology, Tokyo Institute of Technology, Yokohama, Kanagawa 226-8503, Japan; Department of Life Science and Technology, School of Life Science and Technology, Tokyo Institute of Technology, Yokohama, Kanagawa 226-8503, Japan; Institute of Innovative Research, Tokyo Institute of Technology, Yokohama, Kanagawa 226-8503, Japan; Department of Life Science and Technology, School of Life Science and Technology, Tokyo Institute of Technology, Yokohama, Kanagawa 226-8503, Japan

## Abstract

In eukaryotes, Dmc1 and Rad51 are key proteins of homologous recombination. The Swi5–Sfr1 complex in fission yeast, a conserved auxiliary factor, stimulates DNA strand exchange driven by both Dmc1 and Rad51. Interestingly, biochemical analysis suggested that Swi5–Sfr1 regulates strand exchange activities of these recombinases differently, but the mechanisms were unclear. We previously developed a real-time system to analyze Rad51-driven DNA strand exchange and identified two topologically distinct three-stranded intermediates (complex 1 (C1) and complex 2 (C2)). Swi5–Sfr1 facilitates the C1–C2 transition and releases single-stranded DNA (ssDNA) from C2, acting as a strand exchange activator. In this study, we investigated fission yeast Dmc1-driven DNA strand exchange and the role of Swi5–Sfr1 in Dmc1 activity in real-time. Kinetic analysis revealed a three-step model for the Dmc1-driven reaction, similar to that of Rad51. Although Swi5–Sfr1 stimulated the Dmc1-driven reaction, it had a weaker impact than Rad51. Furthermore, Swi5–Sfr1 enhanced the association of Dmc1 with ssDNA by promoting filament nucleus formation, acting as a mediator, unlike its role with Rad51. This stimulation mechanism also differs from that of Ca^2+^ or ATP analog, AMP–PNP. Our findings suggest that Swi5–Sfr1 stimulates strand exchange activities of Dmc1 and Rad51 via different reaction steps.

## Graphical Abstract

**Graphical Abstract:**
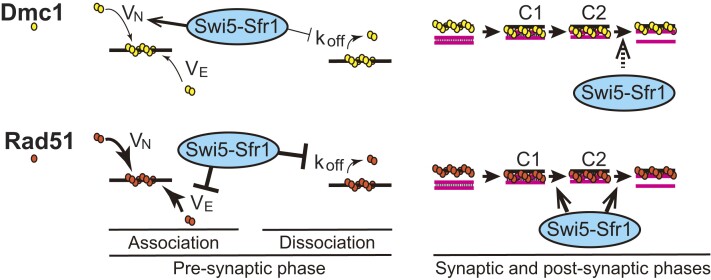

## Introduction

Homologous recombination plays a crucial role in accurate repair of DNA double-strand breaks (DSBs) and the rescue of collapsed DNA replication forks in the mitotic cell cycle. It is also involved in creating genetic diversity and ensuring accurate chromosome segregation during meiosis (1–4). The DNA strand exchange reaction between two homologous sequences is a central step in homologous recombination and is catalyzed by evolutionarily conserved RecA family recombinases (3,5,6). In eukaryotes, there are two RecA family recombinases: Dmc1 and Rad51. Dmc1 specifically functions during meiotic recombination, while Rad51 functions in both mitosis and meiosis (5–7).

In the DNA strand exchange reaction, a recombinase binds to single-stranded DNA (ssDNA) and forms a right-handed helical nucleoprotein complex called a presynaptic filament (3,5,6). This filament captures a donor double-stranded DNA (dsDNA) and forms a three-stranded intermediate that initiates the homology search. Upon encountering a homologous sequence, the recombinase facilitates strand exchange between the recombinase-bound ssDNA and donor dsDNA, generating a new heteroduplex (5–7). Although Rad51 and Dmc1 function in different cell cycles, they have very similar biochemical properties. Both bind to ssDNA and form structurally similar presynaptic filaments, and their DNA strand exchange reactions proceed in an ATP-dependent manner (8–11).

Eukaryotic recombinases require various auxiliary proteins to initiate and complete DNA strand exchange reactions. The formation of DSBs is an initial event in homologous recombination *in vivo*. The DSB ends are resected to generate 3′-overhanging ssDNA regions, which are immediately protected by their binding to replication protein A (RPA). Some auxiliary factors, known as mediators, such as Rad52, Rad51 paralogs and BRCA2, facilitate the displacement of RPA during formation of presynaptic filaments (5,6,12–14).

The Swi5–Sfr1 complex from the fission yeast *Schizosaccharomyces pombe* is an evolutionarily well-conserved auxiliary factor that acts on both Dmc1- and Rad51-promoted DNA exchange (15–17). Swi5–Sfr1 stabilizes presynaptic filaments containing Dmc1 and Rad51 and promotes DNA strand exchange reactions driven by these two recombinases (15,18,19). However, at the molecular level, Swi5–Sfr1 functions differently in Dmc1- and Rad51-driven strand exchange reactions. Swi5–Sfr1 assists Dmc1 in forming filaments on RPA-coated ssDNA as a mediator (19), but it does not assist Rad51 in this context; Rad52 performs this function instead (18). In addition, Swi5–Sfr1 stimulates ATPase activity of Rad51 depending on ssDNA, but not that of Dmc1 (18,19). In mice, SWI5–SFR1 enhances ADP release from the RAD51–ssDNA filament to maintain the filament in active form (20). These differences likely arise from variations in the mechanisms by which Swi5–Sfr1 regulates DNA strand exchange activity driven by Dmc1 and Rad51.

Dmc1 and Rad51 differ in how they use ATP and divalent cations in the DNA strand exchange reaction. The Rad51-driven DNA strand exchange reaction is promoted in the presence of AMP–PNP, a non-hydrolysable ATP analog (21). A human RAD51 mutant proficient in ATP binding but deficient in ATP hydrolysis exhibits higher strand exchange activity than wild-type RAD51 (21,22). Ca^2+^, which attenuates ATP hydrolysis, promotes the DNA strand exchange reaction driven by RAD51 (16,23,24). These results suggest that prevention of accumulation of inactive ADP-bound Rad51, which is produced by the intrinsic ATPase activity of Rad51 in presynaptic filaments, leads to enhance strand exchange activity (16,23,24).

In the case of human DMC1, AMP–PNP and ATP similarly stimulate DNA strand exchange activity (25,26). An ATP hydrolysis-deficient DMC1 mutant does not show higher activity than wild-type DMC1, in contrast to RAD51 (26). Ca^2+^ promotes the DNA strand exchange activity of Dmc1 (25,27). However, the main mode of Ca^2+^ stimulation is not necessarily to prevent the accumulation of the inactive ADP-bound form of DMC1 (25,27). Instead, it is proposed that the stimulation of DMC1 is facilitated presumably through conformational changes induced by the Ca^2+^ ion, which binds to a protein site separate from the Mg^2+^·ATP-binding pocket (25). These results, together with the difference in the Swi5–Sfr1 functions, suggest that the regulatory mechanism of presynaptic filament formation of Dmc1 is distinct from that of Rad51. However, the molecular mechanism underlying these differences remains to be clarified.

Recently, we established a fluorescence resonance energy transfer (FRET)-based real-time monitoring system for the DNA strand exchange reaction, which enables direct analysis of the kinetics of the strand exchange reaction during the synaptic phase (28,29). Using this system, we revealed that the DNA strand exchange reaction driven by fission yeast *S. pombe* Rad51 proceeds in three steps involving two reaction intermediates, complex 1 (C1) and complex 2 (C2), both of which consist of three DNA strands (28,30). C1 comprises the donor dsDNA, which retains the original base pairing, and ssDNA in the presynaptic filament. By contrast, C2 comprises ssDNA in the presynaptic filament intertwined with the complementary strand of the donor dsDNA, leading to formation of a new heteroduplex DNA. Consequently, the three steps are step 1; formation of C1, step 2; transition from C1 to C2 and step 3; release of the non-complementary donor strand from C2. ATP binding of Rad51 is sufficient for C1 formation, while ATP hydrolysis by Rad51 is particularly important for step 3. Swi5–Sfr1 strongly promotes steps 2 and 3, depending on the ATPase activity of Rad51, acting as a strand exchange activator. Ca^2+^ promotes step 2. These results clarify the functions of Swi5–Sfr1 and Ca^2+^ in the synaptic phase, unlike previous studies that focused on the role of these factors in the stabilization of presynaptic filaments (28).

In this study, to clarify the mechanism of the DNA strand exchange reaction driven by *S. pombe* Dmc1 and the regulation of Dmc1 activities by Swi5–Sfr1 and Ca^2+^ during presynaptic and synaptic phase, we performed the FRET-based real-time kinetic analyses along with other methodologies. The results demonstrate that the Dmc1-driven DNA strand exchange reaction proceeds in three steps, similar to the Rad51-driven DNA strand exchange reaction. However, Swi5–Sfr1 promoted only Step 3, and its overall stimulatory effect in the synaptic phase was much weaker during the strand exchange reaction driven by Dmc1 than during that driven by Rad51. Moreover, Swi5–Sfr1 promoted filament nucleation for Dmc1 presynaptic filament formation, acting as a mediator, whereas it inhibits Rad51 presynaptic filament formation. Ca^2+^ promoted Step 1 in Dmc1-driven strand exchange. Ca^2+^ was also more effective in promoting the association and stabilization of Dmc1 presynaptic filaments compared to Swi5–Sfr1 or AMP–PNP, and its effect was more potent than that of Rad51. In summary, this study biochemically clarifies that the DNA strand exchange reaction promoted by Dmc1 proceeds in three-steps, as in Rad51, but the regulation mechanisms of these recombinase activities are very different. In particular, the Swi5–Sfr1 complex regulates different reaction steps by stimulating filament formation of Dmc1 as a mediator and strand exchange by Rad51 as a reaction activator.

## Materials and methods

### Materials

Fission yeast Dmc1, Rad51 and Swi5–Sfr1 were purified as described previously (15,18,19). Protein concentration was determined by measuring the absorbance at 280 nm. The molar extinction coefficients of Dmc1, Swi5–Sfr1 and Rad51 are 1.23 × 10^4^, 1.44 × 10^4^ and 1.86 × 10^4^ M^−1^cm^−1^ respectively. The DNA substrates used in this study were purchased from Eurofins Genomics (Ebersberg, Germany) and are listed in Supplementary Table S1.

### Real-time DNA strand pairing and displacement assays

These assays were conducted as described previously (29). For the DNA pairing assay, Dmc1 protein (2.0 μM) was added to 1.6 ml buffer A (30 mM HEPES–KOH [pH 7.5], 25 mM KCl, 5 mM divalent metal ion [MgCl_2_ or CaCl_2_], 1 mM DTT, 0.1% [w/v] BSA, 0.0075% Tween 20 and 1 mM adenine nucleotide [ATP, ADP or AMP–PNP]) containing 83-mer ssDNA labeled with fluorescein (36 nM, DNA fragment concentration) and incubated at 30°C for 5 min. Swi5–Sfr1was added to the reaction mixture and incubated for a further 5 min. The mixture (1.5 ml) was transferred to a 1.0 × 1.0 cm cuvette in a spectrofluorometer (FP-8300; JASCO, Tokyo Japan). The pairing reaction was started by injecting rhodamine-labeled dsDNA (40, 60 or 83 bp as indicated, each at 36 nM (DNA fragment concentration)) into the reaction mixture using a syringe. Changes in fluorescence of fluorescein were monitored at 525 nm upon excitation at 493 nm. For the displacement assay, the reaction was conducted as described above, except that the reaction volume was 120 μl in a 1.0 × 0.2 cm cuvette and a DNA double-labeled with fluorescein and rhodamine was used. Data were analyzed as described previously (28–30). The maximum FRET efficiency (*E_max_*) values used to convert fluorescence intensities into substrate % or product %, are shown in Supplementary Table S2. All the reaction rates and equilibrium constants simulated by DynaFit are shown in Supplementary Tables S3–S7. Relative changes in equilibrium constants in the presence or absence of Swi5–Sfr1 were calculated using Equation (1) as follows:

(Relative changes in the equilibrium constant) = (Average equilibrium constants of reactions with Swi5–Sfr1, shown in Supplementary Tables S4 or S6)/(Average equilibrium constants of reactions with ATP, shown in Supplementary Tables S4 or S6).(1)

Average equilibrium constants of Rad51 were obtained from a previous study (28).

### DNA-strand-pairing-assay (DSP)- and DNA-strand-displacement-assay (DSD)-initiated abortive DNA strand exchange assays

These assays were initiated essentially using the same procedure as for the pairing assays described above, except that the reaction volume was 120 μl and donor dsDNA was added using a pipette. At 5 min after starting the reaction, 6 μl of 10% SDS was added to collapse the reaction intermediates. Reaction monitoring and data analysis were performed using the same procedure as the DNA strand pairing and displacement assays described above.

C2 and the final product % were calculated using Equations (2) or (3).

For DSP-initiated abortive assay,


(2) \begin{eqnarray*}\begin{array}{@{}l@{}} \left( {{\mathrm{C2\ and\ the\ final\ product}}\,\% } \right) = 100\\ - \left( {{\mathrm{Substrate}}\,\% \,150\ {\mathrm{ sec\ after\ addition\ of\ SDS\ to\ the\ reaction\ mixture}}} \right) \end{array}\end{eqnarray*}

For DSD-initiated abortive assay,


(3) \begin{eqnarray*}\begin{array}{@{}l@{}} \left( {{\mathrm{C2\ and\ the\ final\ product}}\,\,\% } \right) = \\ \left( \begin{array}{@{}l@{}} {\mathrm{Product}}\,\,\% \,\,150\ {\mathrm{ sec\ after\ addition\ of\ }} {\mathrm{SDS\ to\ the\ reaction\ mixture}} \end{array} \right) \end{array}\end{eqnarray*}

C1% were calculated using Equation (4).


(4) \begin{eqnarray*}\begin{array}{@{}l@{}} \left( {{\mathrm{C1\ }}\% } \right) = \left( \begin{array}{@{}l@{}} {\mathrm{Substrate}}\,\,\% \,\ {\mathrm{ just\ before\ adding\ }} {\mathrm{SDS\ to\ the\ reaction\ mixture}}\end{array} \right)\\ - \left( {{\mathrm{C2}}\ {\rm and\ the\ final\ product\,\,}\% \, {\mathrm{ }}} \right) \end{array}\end{eqnarray*}

### DNA pairing assay using ssDNA labeled with 2-aminopurine (2AP)

The experimental procedure was essentially the same as that described previously (30). Dmc1 (2 μM) was mixed into buffer A containing 1 mM ATP and 16A(-)_3 × 2AP, a 2AP-labeled oligo DNA, for 10 min at 30°C. The fluorescence emission of 2AP was monitored at 375 nm upon excitation at 310 nm. Experimental data were analyzed as described previously (30). Fluorescence intensities were converted to the % of substrate considering that the double-stranded DNA formation with Dmc1 quenches the fluorescence to 60.1% compared with the fluorescence of the Dmc1-ssDNA filament.

### Steady-state ssDNA binding assay using fluorescence anisotropy

The steady-state ssDNA binding assay was conducted as described previously (30). Dmc1 or Rad51 proteins were added step-wise to 3 μM (nucleotide concentration) TAMRA-labeled oligo dT in buffer B (30 mM HEPES–KOH [pH 7.5], 100 mM KCl, 3 mM divalent ion [MgCl_2_ or CaCl_2_], 1 mM DTT and 5% glycerol) containing 1 mM adenine nucleotide. After each addition, the reaction mixture was incubated at 25°C for 5 min, and fluorescence anisotropy was measured at 575 nm upon excitation at 546 nm in an FP-8300 spectrofluorometer. The dissociation constant, *K*_*D*_, and Hill coefficient, *n*, were calculated using the Hill equation Equation (5) described below. [recombinase] denotes the recombinase concentration.


(5) \begin{eqnarray*}\begin{array}{@{}l@{}} \left( {{\mathrm{Anisotropy}}} \right) = \left( {{\mathrm{Minimum\ anisotropy}}} \right){\mathrm{ }}\\ + \left\{ {\left( {{\mathrm{Amplitude\ of\ change\ in\ anisotropy}}} \right) \times {{\rm [Recombinase]}}^n} \right\}\\/\left\{ {{T}_{D}^n\ {\mathrm{+ }}\ {{\mathrm{[Recombinase]}}}^n} \right\} \end{array}\end{eqnarray*}

### Real-time Dmc1 or Rad51 filament association and dissociation assays using fluorescence anisotropy

These assays were basically conducted as described previously (30). In the standard association assay, a 1.0 × 1.0 cm cuvette containing 1.5 ml buffer B supplemented with 1 mM adenine nucleotide and 1.2 μM (nucleotide concentration) TAMRA-labeled oligo dT was placed in the spectrofluorometer at 25°C, with continuous stirring at 450 rpm. After 1 min, 75 μl of a protein mixture containing 1 μM Dmc1 or Rad51 with or without Swi5–Sfr1 (Swi5–Sfr1: recombinase ratio from 0.01:1 to 0.5:1) was injected into the reaction. Filament nucleation and elongation velocities, *V_N_* and *V_E_*, were calculated as described elsewhere (31). For Dmc1 and Rad51, *V_N_* was defined by the slope of the reaction within the first 10 and 5 sec post-protein injection, respectively. *V_E_* was defined by the slope at time *T*_1/2_, which was the time point at which half of the ssDNA was occupied by Dmc1 or Rad51. *T*_1/2_ was calculated using Equation (6).


(6) \begin{eqnarray*}\begin{array}{@{}l@{}} \left( {{\mathrm{Anisotropy}}} \right) = \left( {{\mathrm{Minimum\ value\ of\ anisotropy}}} \right){\mathrm{ }}\\ + \left\{ {\left( {{\mathrm{Amplitude\ of\ change\ in\ anisotropy}}} \right) \times {t}^n} \right\}/\left\{ {{T}_{1/2}^n\ {\mathrm{+\ }}{{\mathrm{t}}}^n} \right\} \end{array}\end{eqnarray*}

*V_E_* was calculated as the slope of the anisotropy values obtained from time points of *T*_1/2_ ± 2 sec (total 5 s).

For the dissociation assay, a 1.0 × 1.0 cm cuvette containing 2 ml buffer B supplemented with 1 mM adenine nucleotide (ATP or AMP–PNP) was placed in the spectrofluorometer at 25°C, with continuous stirring at 450 rpm. After 1 min, 50 μl of the reaction mixture from the association assay described above was injected into the cuvette. Fluorescence anisotropy was monitored for 600 (Dmc1) or 1000 (Rad51) sec every 0.5 s. The dissociation rate constant (*k_off_*) was calculated using Equation (7).


(7) \begin{eqnarray*}\begin{array}{@{}l@{}} \left( {{\mathrm{Anisotropy}}} \right) = \left( {{\mathrm{Amplitude\ of\ change\ in\ fluorescence\ anisotropy}}} \right)\\ \times \,{\mathrm{EXP}}\left( { - {k}_{off} \times {\mathrm{t}}} \right) + \left( {{\mathrm{Minimum\ value\ of\ fluorescence\ anisotropy}}} \right) \end{array}\end{eqnarray*}

## Results

### Dmc1-driven DNA strand exchange proceeds in three steps

**Figure 1.**
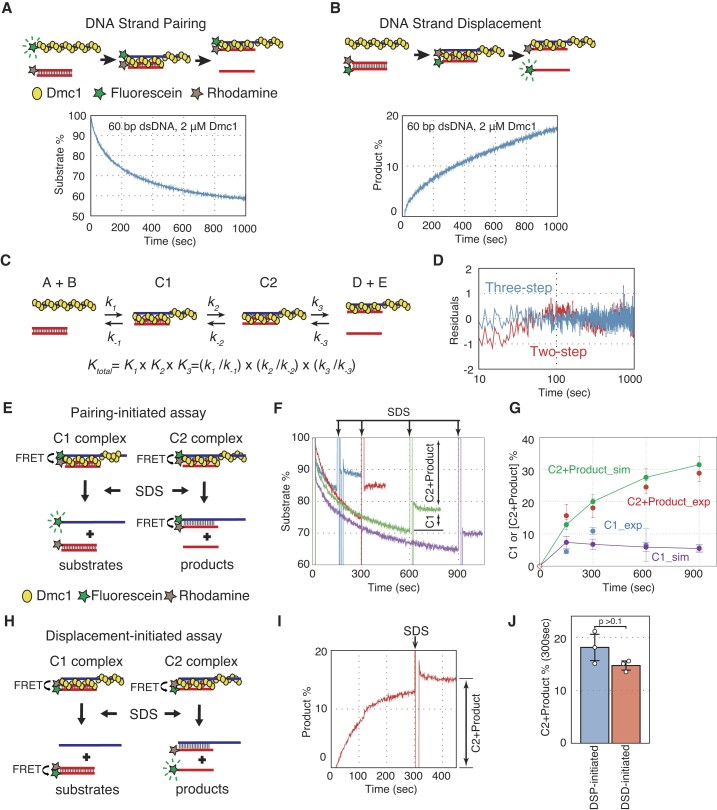
Dmc1-driven DNA strand exchange proceeds through two reaction intermediates containing topologically distinct three-stranded DNA. (**A**) Upper panel: schematics of the FRET-based real-time assay for monitoring DNA strand pairing catalyzed by Dmc1. Dmc1 monomers are represented by yellow circles, while fluorescein and rhodamine are represented by green and brown stars, respectively. Lower panel: time course of DNA strand pairing reactions. Blue, reactions with ATP and Mg^2+^. (**B**) Upper panel: schematic of the FRET-based real-time assay for monitoring DNA strand displacement catalyzed by Dmc1. Lower panel: time course of DNA strand displacement reactions. Blue, reactions with ATP and Mg^2+^. (**C**) Schematic of the three-step reaction model for DNA strand exchange. (A) and (B) represent the presynaptic filament and donor dsDNA, respectively. C1 and C2 represent two distinct reaction intermediates. (D) and (E) represent heteroduplex DNA and ssDNA as the final reaction products, respectively. (**D**) Residuals between experimental data and a theoretical curve simulated using DynaFit for the DNA strand exchange experiment with ATP shown in (A). Blue, residuals between the experimental data and the three-step model; red, residuals between the experimental data and the two-step model. (**E**) Schematic of the DSP-initiated abortive assay. SDS was added to collapse the three-strand intermediate. Emission increases if C1 is converted into substrates, but does not change if C2 is converted into final products. (**F**) Time course for the DSP-initiated abortive assays under the standard condition. SDS was added to the mixture 150 (blue), 300 (red), 600 (green) and 900 (purple) s after initiating the pairing reaction. (**G**) Comparison of the amount of C1 and [C2 + Product] obtained from the results of (F) and the predicted amount of C1 and [C2 + Product] calculated using the reaction rate constants obtained from simulations of the pairing assay of (A). The calculation methods of the C1 and [C2 + Product] from (F) were described in the materials and methods section. Blue, C1 obtained from the results of (F) (shown as C1_exp); red, [C2 + Product] obtained from the results of (F) (shown as C2 + product_exp); purple, the predicted amount of C1 (shown as C1_sim); green, the predicted amount of [C2 + Product] (shown as C2 + product_sim). (**H**) Schematic of the DSD-initiated abortive assay. SDS was added to collapse the three-strand intermediate. Emission increases if C2 is converted into final products, but does not change if C1 is converted into substrates. (**I**) Time courses of the displacement-initiated abortive assays under the standard condition. (**J**) Comparison of the C2 and product % that accumulated in the DSP-abortive and DSD-abortive assays. C2 and product % were obtained from the product % 300 sec after the addition of SDS to the reaction. Blue, DSP-initiated abortive assay; red, DSD-initiated abortive assay. Data shown in (G) and (J) are average values ± s.d. (*n* = 3 independent experiments). Statistical analysis was performed using a two-tailed Student's *t*-test.

To measure the reaction kinetics of the DNA strand exchange reaction driven by *S. pombe* Dmc1, we adapted two FRET-based assays to monitor DNA strand pairing and displacement in real time, which were previously used to monitor Rad51-driven strand exchange (28,29) (Figure 1). In the DNA strand pairing assay, Dmc1 forms presynaptic filaments on fluorescein-labeled ssDNA (Figure 1A). Subsequently, the presynaptic filament is mixed with rhodamine-labeled dsDNA. When the presynaptic filament captures dsDNA to form a three-stranded intermediate, rhodamine quenches the emission of fluorescein by FRET. In the DNA strand displacement assay, the presynaptic filament is mixed with dsDNA doubly labeled with fluorescein and rhodamine (Figure 1B). After forming and processing the three-stranded intermediate, the fluorescein-labeled ssDNA is separated from the rhodamine-labeled DNA strand and emits stronger fluorescence. In both assays, Dmc1 and Swi5–Sfr1 did not affect the emission of fluorescein or the quenching efficiency of fluorescein by rhodamine (Supplementary Figures S1A and B). To determine the concentration of Dmc1 under the standard reaction conditions, which include 5 mM Mg^2+^, 1 mM ATP, 83-mer ssDNA and 60 bp dsDNA (both DNAs were 36 nM, as the fragment concentration), we performed the pairing assay with varying concentrations of Dmc1 (Supplementary Figure S1C). In the presence of 2 μM Dmc1, which was twice the amount required for ssDNA assuming one Dmc1 monomer binds to every three nucleotides, the pairing reaction was most efficient. Under these conditions, the Dmc1–ssDNA filament remained stable for over 1000 s (Supplementary Figure S1D). Thus, we adopted these concentrations of Dmc1 and ssDNA as the standard reaction conditions.

We examined the pairing and displacement activities of Dmc1 under standard reaction conditions (Figures 1A and B). Dmc1 converted 40% of substrates into a three-stranded complex and half of the intermediates into the final product within 1000 s in both assays. To understand the reaction kinetics of DNA strand exchange catalyzed by Dmc1, we simulated the pairing reaction using DynaFit (32), with the assumption that the presynaptic filament is a stable, single molecule (Supplementary Figure S1D), like in the Rad51-driven strand exchange reaction (28). We evaluated two-step and three-step models (Figure 1C) and found that the residuals between the experimental values and simulated values for the three-step model were coincident without systematic deviation, in contrast to the two-step model (Figure 1D).

We further investigated the effect of substrate concentration on the kinetics of the pairing reaction by doubling the concentrations of the Dmc1-ssDNA filament and dsDNA to 72 nM, while maintaining a constant ratio of Dmc1 to ssDNA (Supplementary Figure S1E). The results showed no significant difference in the shape of the reaction curve or the amount of reaction product at 1000 s after the start of the reaction, between the two conditions. Simulation of the pairing assay with higher substrate concentrations using Dynafit indicated that the three-step model fit better than the two-step model (Supplementary Figure S1F). Furthermore, the reaction rate constants obtained from the simulation with the three-step model did not significantly differ between the two conditions (Supplementary Figure S1G).

These clearly indicates that Dmc1-driven DNA strand exchange proceeds in three steps via two distinct three-stranded intermediates, like in the Rad51-driven DNA strand exchange reaction (28).

### The two three-stranded intermediates are topologically distinct

Previously, we demonstrated that Rad51-driven DNA strand exchange proceeds via two topologically distinct three-stranded intermediates, C1 and C2. In C1, the donor dsDNA retains its original base pairing. Conversely, in C2, the ssDNA that was present in the presynaptic filament intertwines with the complementary strand of the donor dsDNA, consequently generating a new heteroduplex (28,30). To examine whether these two intermediates are also formed during Dmc1-driven strand exchange, we attempted to conduct two abortive DNA strand exchange assays (DNA strand pairing-assay [DSP]-initiated and DNA strand displacement-assay [DSD]-initiated abortive assays) (Figures 1E and H), similar to those used to analyze Rad51-driven strand exchange (28). In these abortive assays, SDS was added to the reactions to collapse the three-stranded intermediates, leading to their conversion into the substrates or the final products. C1 and C2 intermediates were expected to be converted into the substrates and final products by SDS, respectively.

In the DSP-initiated abortive assay, an increase in fluorescence emission indicates that the reaction intermediates have collapsed back into substrates, suggesting the presence of C1 in the mixture (Figure 1E). To abort the pairing assay, SDS was added to the mixture 150, 300, 600 or 900 s after the initiation of pairing assay. The amount of C1 and the combined amount of C2 and the final product at each time point were calculated from three independent experiments (Figures 1F and G). The results show that 4–10% of the intermediates were converted into the substrates, and the amounts of the C1 complex changed little over time. In contrast, the combined amounts of C2 and the final product increased over time.

In the DSD-initiated abortive assay, an increase in fluorescence emission indicates the conversion of the reaction intermediates into the final products, suggesting the presence of C2 in the mixture (Figure 1H). After SDS was added to the mixture 300 sec after the initiation of the displacement assay, ∼2.5% of the intermediates were converted into the final products (Figure 1I). The combined amounts of C2 and the final products, determined from three independent pairing- and displacement-assay-initiated abortive assays, were not statistically significantly different (*P* > 0.1; Figure 1J). This indicates that both the pairing assay and the displacement assay are monitoring the same reaction process from different perspectives. In conclusion, these results suggest that Dmc1-driven DNA strand exchange proceeds via two topologically distinct intermediates, C1 and C2, as expected.

Furthermore, the kinetics of the two intermediates and the final product were examined for consistency with the three-step model. For this purpose, the amounts of C1 and the combined amounts of C2 and the final products at 150, 300, 600 and 900 s after starting the reaction were predicted using the reaction rate constants obtained from the simulation of the pairing assay in Figure 1A with the three-step model. The predicted values matched very well with those obtained from the DSP-initiated abortive assay described above (Figure 1G), further supporting the validity of the three-step model. These results reinforce the three-step model containing the two topologically distinct intermediates.

### Nucleotide cofactor dependence of Dmc1-driven DNA strand exchange reaction

**Figure 2.**
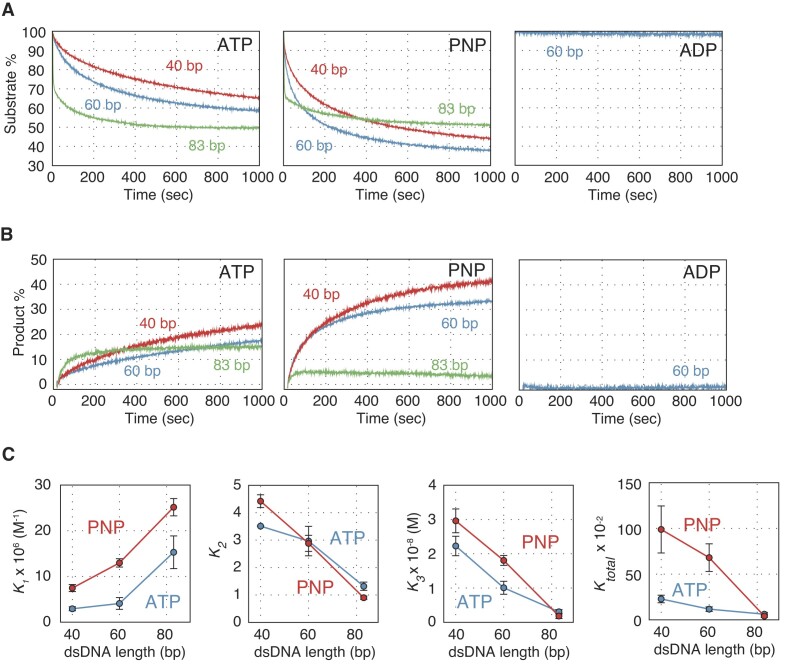
The impact of ATP binding and hydrolysis on Dmc1-driven DNA strand exchange reaction. (**A**) DNA strand pairing reactions with various lengths of donor dsDNA. Left panel: results from reactions containing ATP (1 mM). Center panel: results from reactions containing AMP–PNP (1 mM). Right panel: results from reactions containing ADP (1 mM). Blue, reactions initiated with 40 bp dsDNA; red, reactions initiated with 60 bp dsDNA; green, reactions initiated with 83 bp dsDNA. (**B**) DNA strand displacement reactions with various lengths of donor dsDNA. Left panel, results of reactions containing ATP (1 mM); center panel, results of reactions containing AMP–PNP (1 mM); right panel: results of reactions containing ADP (1 mM). Blue, reactions initiated with 40 bp dsDNA; red, reactions initiated with 60 bp dsDNA, and green, reactions initiated with 83 bp dsDNA. (**C**) *K_total_*, *K*_1_, *K*_2_ and *K*_3_ were calculated using reaction rate constants obtained from the simulation in (A). Blue, reactions containing ATP; red, reactions containing AMP–PNP. Data shown in (C) are average values ± s.d. (*n* = 3 independent experiments).

To examined the impact of adenine nucleotide on Dmc1-driven DNA strand exchange reaction, we conducted that DNA strand pairing and displacement assay initiated by 40, 60 and 83 bp dsDNA in the presence of ATP, ADP or a non-hydrolysable analog of ATP, AMP–PNP (Figure 2). In the presence of ATP, the DNA pairing reaction was faster with longer donor dsDNA (Figure 2A, left), whereas the DNA displacement reaction was slightly slower with longer donor dsDNA (Figure 2B, left). These results suggest that the presynaptic filament composed of Dmc1 and ssDNA forms a three-stranded intermediate more efficiently with longer donor dsDNA and that the processing of these intermediates is more inefficient than the intermediate with shorter dsDNA, as observed previously for the Rad51-driven reaction (28).

Dmc1 failed to form any intermediate or final product in reactions with ADP (Figures 2A, right and B, right). Surprisingly, AMP–PNP attenuated pairing and displacement reactions with 83 bp donor dsDNA compared with ATP, whereas it stimulated these reactions with shorter dsDNA (Figures 2A, center and B, center). These results indicate that the ATP-bound form of Dmc1 is sufficient for three-stranded intermediate formation. Although ATP hydrolysis is unnecessary for the completion of DNA strand exchange with short dsDNA, Dmc1 cannot complete strand exchange without ATP hydrolysis with long DNA, such as the 83 bp dsDNA donor substrate used in the reactions described above. The impact of ATP hydrolysis by Dmc1 was also tested by the three-strand exchange assay using plasmid-sized DNA substrates (Supplementary Figures S2A–C). In the presence of ATP, the substrates were converted into the final product (∼12%) and the intermediates (∼57%). In the presence of AMP–PNP, although Dmc1 could produce as many reaction intermediates as in the presence of ATP, few final products were detected. These results were consistent with the DNA pairing and displacement assays.

To obtain quantitative parameters for the Dmc1-driven strand exchange reaction, we simulated the results of the DNA pairing assay (Supplementary Figure S3 and Supplementary Table S3). The simulations provided reaction equilibrium constants for each reaction step (*K*_1_*= k*_1_*/k*_-1_, *K*_2_ = *k*_2_/*k*_-2_ and *K*_3_ = *k*_3_/*k*_-3_). The equilibrium constant for the total reaction (*K_total_*) was defined as *K*_1_ × *K*_2_ × *K*_3_(Figure 2C).

In the presence of ATP, *K*_1_ values increased in a dsDNA length-dependent manner, while *K*_2_, *K*_3_ and *K_total_* values decreased, indicating that the Dmc1 presynaptic filament is more likely to capture long dsDNA, but that the subsequent steps leading to the formation of the final products are less likely to proceed, as observed previously for the Rad51-driven reaction (28).

Interestingly, the *K*_1_ values were higher in reactions with AMP–PNP than in those with ATP for the three dsDNAs. On the other hand, *K*_2_ values with ATP did not differ from those with AMP–PNP. Conversely, the *K*_3_ values with AMP–PNP were higher than those with ATP in the reactions containing 40 or 60 bp dsDNAs, whereas they were smaller and similar between ATP and AMP–PNP in reactions containing 83 bp dsDNA. The *K_total_* values were much higher for AMP–PNP than for ATP when short dsDNAs (40 or 60 bp) were used, but these values were similarly lower for both ATP and AMP–PNP in the reaction with 83 bp dsDNA, mirroring the pattern seen with *K*_3_.

Taken together, the results indicate that ATP binding to the Dmc1 presynaptic filament stimulates step 1 of C1 intermediate formation regardless of DNA length. Additionally, there is a threshold length of donor dsDNA that requires ATP hydrolysis at step 3, when the C2 intermediate is converted to the final product. This suggests that ATP hydrolysis by Dmc1 is essential for expanding the heteroduplex DNA region.

### Swi5–Sfr1 complex stimulates step 3 of Dmc1-driven DNA strand exchange in the presence of ATP hydrolysis

**Figure 3.**
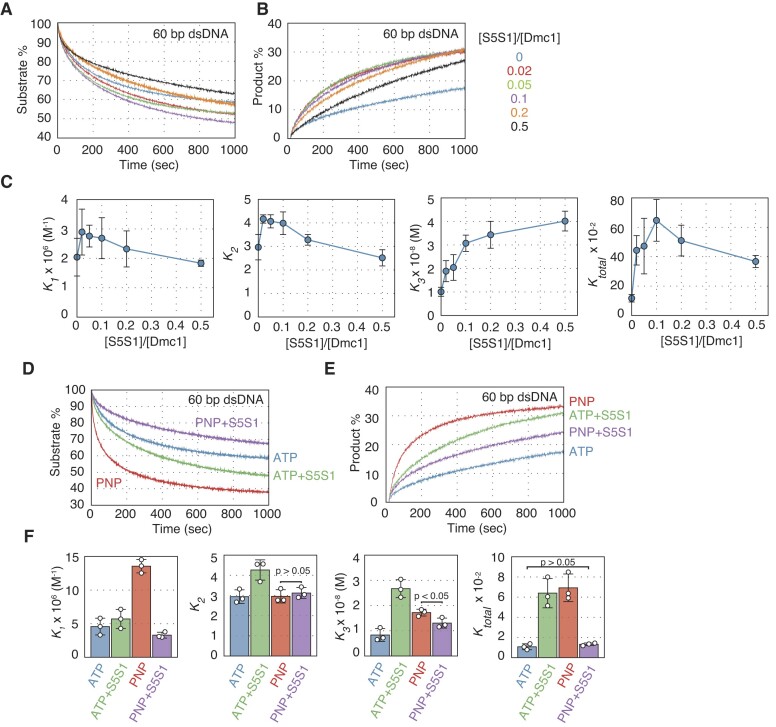
Stimulatory effects of Swi5–Sfr1 on Dmc1-driven DNA strand exchange. Time course of DNA strand pairing (**A**) and DNA strand displacement reactions (**B**). The reaction mixtures contained Dmc1 (2 μM) and Swi5–Sfr1 at various Swi5–Sfr1:Dmc1 ratios. (**C**) *K_total_*, *K*_1_, *K*_2_ and *K*_3_ were calculated using reaction rate constants obtained from simulations of the reactions in (A). Time course of DNA strand pairing (**D**) and DNA strand displacement (**E**) reactions, respectively, with ATP or AMP–PNP with or without Swi5–Sfr1 (0.2 μM). (**F**) *K_total_*, *K*_1_, *K*_2_ and *K*_3_ values were calculated using reaction rate constants obtained from simulations of the reactions in (D). Blue, reactions with ATP; red, reactions with AMP–PNP (shown as PNP); green, reactions with ATP and Swi5–Sfr1; purple, reactions with AMP–PNP and Swi5–Sfr1. Data shown in (C) and (F) are average values ± s.d. (*n* = 3 independent experiments). Statistical analysis was performed using a two-tailed Student's *t*-test.

To examine the impact of the Swi5–Sfr1 complex on Dmc1-driven DNA strand exchange, we conducted a real-time assay in the presence of various concentrations of Swi5–Sfr1 complex (Swi5–Sfr1:Dmc1 ratios of 0.002:1 to 0.1:1) using 60 bp dsDNA. At low concentrations of Swi5–Sfr1 (Swi5–Sfr1:Dmc1 ratios of 0.02:1 to 0.1:1), both DNA pairing and displacement were enhanced in a concentration-dependent manner (Figure 3A and B). Maximum stimulation occurred at a ratio of Swi5–Sfr1 to Dmc1 of 0.1:1. At higher Swi5–Sfr1:Dmc1 ratios (0.2:1 and 0.5:1), the velocities of both DNA pairing and displacement were lower than those at the ratio of 0.1:1. These results are consistent with previous biochemical analyses using plasmid-sized DNA substrates (15,19).

Simulation of the three-step model was used to obtain reaction equilibrium constants for the pairing assay (Figure 3C, Supplementary Figure S3 and Supplementary Table S4). The *K_1_* values were roughly similar at all concentrations of Swi5–Sfr1. Although *K_1_* values increased slightly at Swi5–Sfr1:Dmc1 ratios of 0.02:1 to 0.1:1, the values were not statistically different from that without Swi5–Sfr1. This characteristic clearly distinguishes step 1 of the Dmc1 reaction from that of the Rad51 reaction, where C1 formation by Rad51 is strongly inhibited by Swi5–Sfr1 in a concentration-dependent manner (28).

The *K*_2_ value, an indicator of step 2 (the C1–C2 intermediate transition), of the Dmc1-driven strand exchange reaction was slightly increased (∼1.3 fold) by Swi5–Sfr1 at Swi5–Sfr1:Dmc1 ratios of 0.02:1 to 0.1:1, but was decreased at higher ratios. The *K*_3_, value, an indicator of step 3 (final products formation), was increased by Swi5–Sfr1 in a Swi5–Sfr1 concentration-dependent manner, and was approximately 4-fold larger when the ratio was 0.5:1. The *K_total_* value, an indicator of overall progression, also increased in a Swi5–Sfr1 concentration-dependent manner with a peak (∼6-fold) at a Swi5–Sfr1:Dmc1 ratio of 0.1:1.

We next examined whether ATP hydrolysis by Dmc1 was required for its stimulation by Swi5–Sfr1 (Figures 3D–F). In reactions containing Swi5–Sfr1 and AMP–PNP, both pairing and displacement by Dmc1 were dramatically reduced (Figures 3D and E). Kinetic analysis indicated that Swi5–Sfr1 decreased the *K*_1_ values of reactions containing AMP–PNP, leading to a decrease in the *K_total_* value (Figure 3F, Supplementary Figure S3 and Supplementary Table S5). The results indicate that ATP hydrolysis by Dmc1 is critical for the stimulatory function of Swi5–Sfr1 and that stimulation by AMP–PNP occurs via a mechanism that is distinct from that of Swi5–Sfr1.

### Swi5–Sfr1 stimulates Dmc1-driven DNA strand exchange independently of donor dsDNA length

**Figure 4.**
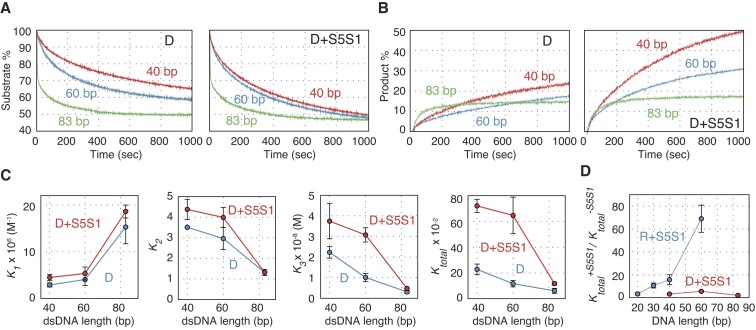
Stimulatory effects of Swi5–Sfr1 on Dmc1-driven DNA strand exchange with donor dsDNA of various lengths. (**A**) DNA strand pairing reactions with donor dsDNA of various lengths (40, 60or 83 bp). Left panel, Dmc1-only reactions; right panel, reactions with Dmc1 (2 μM) and Swi5–Sfr1 (0.2 μM). (**B**) DNA strand displacement reactions with donor dsDNA of various lengths (40, 60 or 83 bp). Left panel, Dmc1-only reactions. Right panel, reactions with Dmc1 (2 μM) and Swi5–Sfr1 (0.2 μM). Red, reactions initiated with 40 bp dsDNA; blue, reactions initiated with 60 bp dsDNA; green, reactions initiated with 83 bp dsDNA. (**C**) *K_total_*, *K*_1_, *K*_2_ and *K*_3_ were calculated using reaction rate constants obtained from the simulation of the reactions in (A). Blue, Dmc1-only reactions; red, reactions with Dmc1 (2 μM) and Swi5–Sfr1 (0.2 μM). (**D**) Changes in relative values of *K_total_* (reactions with Swi5–Sfr1/recombinase-only reactions) for various donor DNA lengths. Relative changes in *K_total_* in Rad51-driven reactions were calculated using data published in Ito *et al.* (28), as described in Materials and Methods. Blue, reactions containing Rad51; red. reactions containing Dmc1. Data shown in (C) and (D) are mean ± standard deviation (*n* = 3 independent experiments).

We previously reported that the stimulation of the Rad51 strand exchange reaction by Swi5–Sfr1 (represented as the ratios of reaction constants ± Swi5–Sfr1) is more pronounced in reactions containing long donor dsDNA (28). This was particularly evident in the final step 3, suggesting that Swi5–Sfr1 facilitates the expansion of the heteroduplex region by stimulating the completion of the reaction (28). To investigate whether Swi5–Sfr1 activates Dmc1 through a mechanism similar to that of Rad51, we performed pairing and displacement assays using donor dsDNA of varying lengths (40 and 83 bp dsDNA) with Swi5–Sfr1 complex (Swi5–Sfr1:Dmc1 ratio of 0.1:1) (Figures 4A and B). In the pairing assay, Swi5–Sfr1 stimulated Dmc1 to form three-stranded intermediates using 40 and 60 bp donor dsDNA. However, DNA pairing with an 83 bp donor dsDNA was not stimulated by Swi5–Sfr1 (Figure 4A). Likewise, Dmc1-driven DNA displacement was enhanced by Swi5–Sfr1 in reactions with 40 or 60 bp dsDNA, but not in reactions with 83 bp donor DNA (Figure 4B).

To elucidate the effect of Swi5–Sfr1 on the aforementioned reaction, we analyzed the results of the pairing assay using a three-step model (Figure 4C, Supplementary Figure S3 and Supplementary Table S6). *K*_1_ and *K*_2_ values increased and decreased, respectively, in a DNA length-dependent manner, regardless of the presence of Swi5–Sfr1. In the presence of Dmc1 alone, *K*_3_ values decreased linearly in a duplex DNA length-dependent manner. Although the *K*_3_ value did not change significantly between reactions with 40 and 60 bp dsDNA (the *K*_3_ value with 60 bp dsDNA was ∼83% of that of 40 bp dsDNA), the *K*_3_ value decreased substantially when the dsDNA length was changed from 60 to 83 bp (the *K*_3_ value with 83 bp dsDNA was ∼16% of that of 40 bp dsDNA). *K_total_* values decreased similarly to the *K*_3_ values. Notably, the *K*_3_ and *K_total_* reduction in the presence of Swi5–Sfr1 is similar to the reduction observed in the presence of AMP–PNP.

In contrast to Rad51, the stimulatory effect of Swi5–Sfr1 on Dmc1 was independent of dsDNA length and was much weaker, indicating that the mechanism responsible for stimulatory activity of Swi5–Sfr1 differs between the two recombinases (Figure 4D).

### Ca^2+^ stimulates C1 intermediate formation but impairs C2 transition

**Figure 5.**
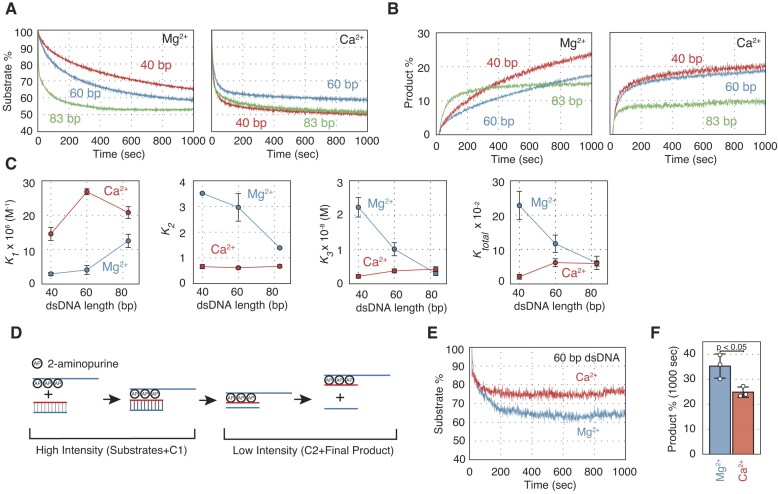
Ca^2+^ strongly stimulates the formation of the initial three-strand intermediate C1 but inhibits the C1–C2 transition during the Dmc1-driven strand exchange reaction. (**A**) DNA strand pairing reactions with donor dsDNA of various lengths (40, 60 or 83 bp). Left panel, reactions in the presence of Mg^2+^ (5 mM). Right panel, reactions in the presence of Ca^2+^ (5 mM). (**B**) DNA strand displacement reactions with donor dsDNA of various lengths (40, 60 or 83 bp). Left panel, reactions in the presence of Mg^2+^ (5 mM). Right panel, reactions in the presence of Ca^2+^ (5 mM). Blue, reactions initiated with 40 bp dsDNA; red, reactions initiated with 60 bp dsDNA; green, reactions initiated with 83 bp dsDNA. (**C**) *K_total_*, *K*_1_, *K*_2_ and *K*_3_ were calculated using reaction rate constants obtained from the simulation of the reactions in (A). (**D**) Schematic of the DNA strand exchange reaction using ssDNA containing 2AP. (**E**) Time course of the DNA strand pairing reaction containing Mg^2+^ (5 mM) or Ca^2+^ (5 mM). Blue, reactions containing Mg^2+^; red, reactions containing Ca^2+^. (**F**) Change in fluorescence intensity of 2AP 1000 s after initiation of the pairing reaction, calculated from (E). Blue, reactions containing Mg^2+^; red, reactions containing Ca^2+^. Data shown in (C) and (F) are mean ± standard deviation (*n* = 3 independent experiments). Statistical analysis was performed using a two-tailed Student's *t*-test.

Ca^2+^ is a well-known activator of eukaryotic recombinases, including Dmc1 and Rad51 (23,25,27,33). We previously reported that Ca^2+^ stimulates the C1–C2 transition in Rad51-driven DNA strand exchange reactions (28). Thus, we investigated the mechanism via which Ca^2+^ stimulates Dmc1-driven DNA strand exchange in pairing and displacement assays (Figures 5A–C).

The pairing assays demonstrated that intermediate formation velocities were much faster in the presence of Ca^2+^ than in the presence of Mg^2+^ irrespective of dsDNA length, and the reaction reached a plateau 200 s after the reaction started (Figure 5A). The displacement assays also showed that final product formation was faster with Ca^2+^ than with Mg^2+^, reaching a plateau after 400 s under all conditions. However, the amount of the final product was higher in reactions containing Mg^2+^ under all conditions (Figure 5B). These results were consistent with the three-strand exchange assay using plasmid-sized DNA substrates (Supplementary Figures S2A–C).

To uncover how Ca^2+^ promotes Dmc1-driven DNA strand exchange, we analyzed the results of pairing assays using the three-step model (Figure 5C, Supplementary Figure S3 and Supplementary Table S7). The *K*_1_ values with Ca^2+^ was greater than those with Mg^2+^ under all conditions. Interestingly, although Ca^2+^ stimulates the C1–C2 transition by Rad51, the *K*_2_ values of Dmc1 reactions were significantly lower with Ca^2+^ than with Mg^2+^ and remained almost constant regardless of dsDNA length. When the reaction was initiated with 40 or 60 bp dsDNA, the *K*_3_ values with Ca^2+^ were significantly lower than those with Mg^2+^. However, in reactions initiated with 83 bp dsDNA, the *K*_3_ values were not statistically different between reactions with Ca^2+^ and those with Mg^2+^. These results show that Ca^2+^ strongly promotes the C1 intermediate formation step, but is less effective in promoting the C1–C2 intermediate transition and final product formation than Mg^2+^, leading to the accumulation of C1 intermediates in the reaction.

To examine whether the C1-C2 intermediate transition is less likely to proceed in the presence of Ca^2+^, we performed a pairing assay using 2-aminopurine (2AP), a fluorescent analog of adenine that forms base pairs with thymine (Figures 5D–F). Since the fluorescence of 2AP is quenched when it forms base pairs with thymine, it is possible to detect the formation of the C2 intermediate specifically, which contains newly generated heteroduplex with 2AP labeled ssDNA (30,34). The results showed that C2 intermediate formation was greater in reactions with Mg^2+^ (∼35%) than in those with Ca^2+^ (∼25%).

The results demonstrate that Ca^2+^ promotes the formation of the initial intermediate C1 in Dmc1-driven DNA strand exchange. However, Ca^2+^ impeded the transition between intermediates, and late intermediate formation was less efficient than in reactions containing Mg^2+^. Ca^2+^ may not only attenuate the ATP hydrolysis activity of Dmc1 but also alter the properties of the Dmc1 filament, because the mechanism of stimulation of Dmc1 by Ca^2+^ was clearly different from that of stimulation by AMP–PNP. Interestingly, the activation mechanisms of Dmc1 and Rad51 by Ca^2+^ were different, in agreement with previous studies on their human counterparts (23,25).

### AMP–PNP, Swi5–Sfr1 and Ca^2+^ stimulate ssDNA binding of Dmc1

**Figure 6.**
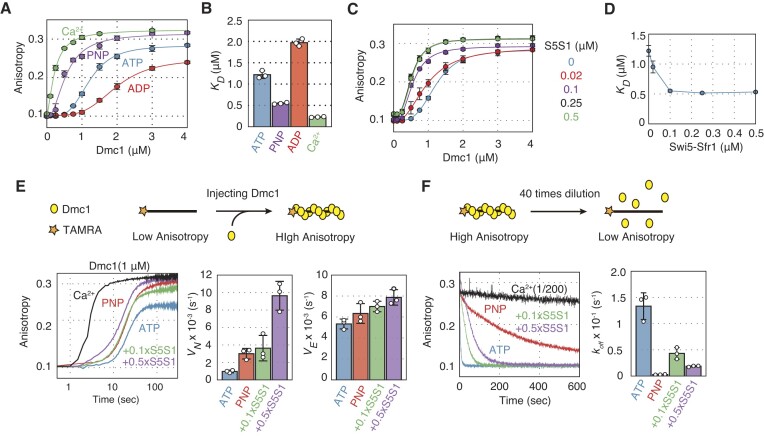
AMP–PNP, Swi5–Sfr1 and Ca^2+^ stimulate the ssDNA binding activity of Dmc1 through different mechanisms. (**A**) The effects of adenine nucleotides and Ca^2+^ on the affinity of Dmc1 for ssDNA measured by fluorescence anisotropy. Dmc1 protein was titrated into reactions containing TAMRA-labeled ssDNA and ATP, ADP, AMP–PNP, or Ca^2+^. (**B**) The dissociation constant, *K_D_*, of Dmc1 from ssDNA was calculated using the Hill equation for each condition. Blue, reactions with ATP; purple, reactions with AMP–PNP; red, reactions with ADP; green, reactions with Ca^2+^. (**C**) The effects of Swi5–Sfr1 on Dmc1 affinity for ssDNA measured using fluorescence anisotropy. Dmc1 protein was titrated into reactions containing TAMRA-labeled ssDNA with various concentrations of Swi5–Sfr1. (**D**) The *K_D_* values of each condition were calculated using the Hill equation. (**E**) Upper panel, schematic of real-time Dmc1 association with ssDNA measured using fluorescence anisotropy. Lower left panel, results of these association assays. Lower right panels, Nucleation and elongation velocities, *V_N_* and *V_E_*, of Dmc1 on ssDNA for each reaction condition. Blue, reactions containing ATP; red, reactions containing AMP–PNP; green, reactions containing Swi5–Sfr1 at 0.1 × the concentration of Dmc1; purple, reactions containing Swi5–Sfr1 at 0.5 × the concentration of Dmc1; black, reactions containing Ca^2+^. (**F**) Upper panel: Schematic of the real-time Dmc1–ssDNA filament dissociation measured using fluorescence anisotropy. Lower left panel, results of these dissociation assays. Lower right panel, dissociation rate constants of Dmc1 from ssDNA for each reaction condition. Data shown in (B)–(F) are average values ± s.d. (*n* = 3 independent experiments). Statistical analysis was performed using a two-tailed Student's *t*-test.

Since AMP–PNP, Swi5–Sfr1 and Ca^2+^ altered the DNA strand exchange activity of Dmc1 through different mechanisms (Figures 1–5), we investigated whether these factors have different effects on the ssDNA binding of Dmc1. We measured the steady-state ssDNA binding activity of Dmc1 using fluorescence anisotropy (Figures 6A–D). The affinities of Dmc1 for ssDNA varied drastically depending on the adenine nucleotide. The *K_D_* value of the reaction with ADP (1.97 μM) was 1.6-fold larger than that of the reaction with ATP (1.22 μM), while the *K_D_* value (0.547 μM) of the reaction with AMP–PNP was approximately half that of the reaction with ATP (Figures 6A and B). Interestingly, in the presence of Ca^2+^, the *K_D_* values (0.227 μM) were even smaller and the affinity of Dmc1 for ssDNA was stronger than in the presence of AMP–PNP.

To examine the effect of Swi5–Sfr1 on the binding of Dmc1 to ssDNA, Dmc1 was titrated into a reaction in which single-stranded DNA had been preincubated with Swi5–Sfr1. The results showed that the addition of 0.1 μM or more of Swi5–Sfr1 resulted in a *K_D_* value (∼0.510 μM) that was about half that of the reaction with Dmc1 alone, and almost the same as that in the presence of AMP–PNP (Figures 6C and D).

The effects of these factors were also analyzed by electrophoresis mobility shift assay (Supplementary Figure S4). The results show that in the presence of AMP–PNP or Ca^2+^, the ssDNA was stacked in the well, suggesting that these factors make the Dmc1 filament stable and stiff. These findings are consistent with the DNA binding assay using fluorescence anisotropy (Figures 6A and B). In the presence of Swi5–Sfr1, the band was more broadly spread to the high molecular weight side, indicating that Dmc1 binding to ssDNA in the presence of Swi5–Sfr1 is stimulated. However, we did not observe ssDNA stacked in the wells, suggesting a different filament quality compared to Ca^2+^ or AMP–PNP. This observation aligns with the anisotropy assay, providing additional information that Swi5–Sfr1 promotes filament assembly of Dmc1, but has weaker stabilizing activity than the other factors (Figures 6E and F).

### AMP–PNP and Swi5–Sfr1 specifically promote the filament nucleation step, while Ca^2+^ promotes both filament nucleation and elongation

To further investigate the effects of AMP–PNP, Swi5–Sfr1 and Ca^2+^ on ssDNA binding activity of Dmc1, we analyzed the association of Dmc1 with the ssDNA filament in real time by measuring fluorescence anisotropy (Figure 6E). The binding curve of Dmc1 to ssDNA was sigmoidal and exhibited two distinct binding phases: an initial, slow binding phase (∼10 s) and a second, rapid binding phase. The initial phase represents the formation of filament nuclei on ssDNA, which is the rate-limiting step of filament formation, while the second phase represents Dmc1–ssDNA filament elongation.

To understand the association kinetics of Dmc1 on ssDNA, we calculated the nucleation and elongation velocities, *V_N_* and *V_E_*, respectively (Figure 6E). *V_N_*, is the slope of the initial 10 sec, and *V_E_*, is the slope of the time (*T_1/2_*) for the anisotropy value to reach half of its highest value. Under the standard conditions containing Mg^2+^ and ATP, *V_N_*, *T_1/2_*, and *V_E_* were 0.966 × 10^−3^ (s^−1^), 20.7 (s), and 5.55 × 10^−3^ (s^−1^), respectively. AMP–PNP stimulated the association of Dmc1 with ssDNA and increased the value of *V_N_* (3-fold) but did not affect the value of *V_E_* compared with the reaction with ATP. This indicates that AMP–PNP specifically stimulates filament nucleation of Dmc1 on ssDNA. Swi5–Sfr1 stimulated the association reaction in a concentration-dependent manner. By calculating the velocities of the two reaction phases, we found that Swi5–Sfr1 also specifically enhanced the filament nucleation step of Dmc1 on ssDNA. Interestingly, when Dmc1 was mixed with Swi5–Sfr1 at half the concentration of Dmc1, the stimulation of the nucleation step was much stronger than under the condition in the presence of AMP–PNP. In the presence of Ca^2+^, Dmc1 binding to ssDNA was noticeably faster than under other conditions. Indeed, we could not calculate *V_N_*in the presence of Ca^2+^_,_ because the filament formation rate was too rapid to distinguish between the two binding phases. The *T_1/2_* value in the reaction with Ca^2+^ was 7-fold lower than that in the presence of Mg^2+^. The shape of the binding curve in the reaction with Ca^2+^ was markedly different from that in the reaction with Mg^2+^ and AMP–PNP, suggesting that the effects of Ca^2+^ on Dmc1 nucleation do not only involve the inhibition of Dmc1 ATP hydrolysis to maintain the ATP-bound state of Dmc1.

### Ca^2+^ substantially stabilizes Dmc1-ssDNA filament

We further investigated the impact of divalent cations, adenine nucleotides, and Swi5–Sfr1 on Dmc1 filament stability. To monitor the dissociation of Dmc1 from the ssDNA filament in real time, we diluted the reaction mixture containing the Dmc1 filament 40-fold in protein- or DNA-free reaction solutions and measured changes in fluorescence anisotropy (Figure 6F). In the presence of ATP and Mg^2+^, the dissociate rate constant (*k_off_*) was 0.133 (s^−1^). The *k_off_* value with AMP–PNP was 46-fold lower than that with ATP, indicating that AMP–PNP strongly stabilizes the Dmc1–ssDNA filament. Although the Dmc1 filament was stabilized 3- and 7-fold at Swi5–Sfr1:Dmc1 ratios of 0.1:1 and 0.5:1, respectively, the stabilizing effect was considerably weaker than that of AMP–PNP. Remarkably, in a reaction containing Ca^2+^, Dmc1 filaments were extremely stable and did not collapse even after 200-fold dilution, further suggesting that Ca^2+^ does not merely inhibit the ATP hydrolysis activity of Dmc1.

### Swi5–Sfr1 and Ca^2+^ regulate Dmc1- and Rad51-ssDNA filament different mechanisms

**Figure 7.**
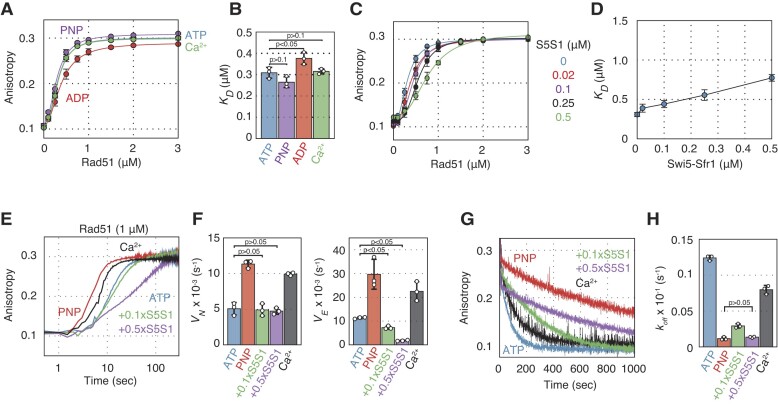
Effects of Swi5–Sfr1, AMP–PNP or Ca^2+^ on Rad51-ssDNA association and dissociation. (**A**) The effects of adenine nucleotides and Ca^2+^ on Rad51 affinity for ssDNA monitored using fluorescence anisotropy. Rad51 protein was titrated into reactions containing TAMRA-labeled ssDNA and ATP, ADP, AMP–PNP or Ca^2+^. (**B**) The dissociation constant, *K_D_*, of Rad51 from ssDNA was calculated using the Hill equation for each condition. Blue, reactions with ATP; purple, reactions with AMP–PNP; red, reactions with ADP; green, reactions with Ca^2+^. (**C**) The effects of Swi5–Sfr1 on the Rad51 affinity for ssDNA measured using fluorescence anisotropy. Rad51 protein was titrated into reactions containing TAMRA-labeled ssDNA with various concentrations of Swi5–Sfr1. (**D**) The *K_D_* values of each condition were calculated using the Hill equation. (**E**) Time courses of real-time association assays of Rad51 binding to ssDNA. (**F**) The nucleation and elongation velocities, *V_N_* and *V_E_*, of Rad51 on ssDNA for each reaction condition. Blue, reactions containing ATP; red, reactions containing AMP–PNP; green, reactions containing Swi5–Sfr1 at 0.1 × concentration of Rad51; purple, reactions containing Swi5–Sfr1 at 0.5 × concentration of Rad51; black, reactions containing Ca^2+^. (**G**) Time course of Rad51 dissociation from ssDNA. (**H**) Dissociation rate constants of Rad51 from ssDNA for each reaction condition. Data shown in (B), (D), (F) and (H) are average values ± s.d. (*n* = 3 independent experiments). Statistical analysis was performed using a two-tailed Student's *t*-test.

Previously, we reported the mechanisms responsible for Rad51-driven DNA strand exchange by Ca^2+^ and Swi5–Sfr1 (28). Since the effects of the two factors on Dmc1 or Rad51 were distinctively different, we examined the effects of Swi5–Sfr1 or Ca^2+^ on the ssDNA binding activity of Rad51 (Figures 7A–D). In the presence of Mg^2+^ and ATP, the *K_D_* value of Rad51 for ssDNA (0.308 μM) was ∼one-quarter of that of Dmc1 (1.22 μM). Unlike Dmc1, the *K_D_* of Rad51 for ssDNA was not noticeably altered by adenine nucleotides or Ca^2+^. However, Swi5–Sfr1 reduced the ssDNA binding affinity of Rad51 in a concentration-dependent manner.

We also examined the effects of Swi5–Sfr1 and Ca^2+^ on the association and dissociation of the Rad51-ssDNA filament (Figures 7E to H). In an association assay, the binding curve of Rad51 to ssDNA displayed a sigmoid shape (Figures 7E). The *V_N_* and *V_E_* of the Rad51 reaction with Mg^2+^ and ATP were 5-fold and 2-fold larger than those of Dmc1, respectively, and in particular, the nucleation step of Rad51 on ssDNA was faster than that of Dmc1, consistent with previous work on budding yeast recombinases (35). AMP–PNP stimulated both nucleation (2.2-fold) and elongation (2.6-fold) of the Rad51-ssDNA filament (Figures 7E and F), while it only enhanced Dmc1-filament nucleation (Figures 6E). Swi5–Sfr1 strongly inhibited Rad51 filament formation in a concentration-dependent manner, and specifically attenuated the filament elongation step (Figures 7E and F), but not Dmc1 filament elongation (Figure 6E). Although Ca^2+^ also stimulated Rad51 filament formation and increased both *V_N_* and *V_E_* 2-fold, the strength of the stimulation by Ca^2+^ was similar to that of AMP–PNP (Figures 7E and F), unlike in the case of Dmc1.

In a dissociation assay, the Rad51-ssDNA filament dissociated slower than the Dmc1-ssDNA filament in reactions with Mg^2+^and ATP, and the *k_off_* of Rad51 under this condition was 10-fold lower than that of Dmc1 (Figures 6F, 7G and H). AMP–PNP significantly stabilized the Rad51 filament, decreasing *k_off_* by 10-fold compared with that of the reaction with ATP. Notably, the *k_off_* values of Dmc1 and Rad51 with AMP–PNP were comparable. Swi5–Sfr1 also stabilized the Rad51 filament in a concentration-dependent manner. Although the stabilization effect of Swi5–Sfr1 on the Dmc1-ssDNA filament was much weaker than that of AMP–PNP, a high amount of Swi5–Sfr1 was as effective in stabilizing the Rad51 filament as AMP–PNP. We could not dissociate the Dmc1-ssDNA filament in the presence of Ca^2+^, while Ca^2+^ slightly stabilized the Rad51-ssDNA filament, but this effect of Ca^2+^ was much weaker than those of AMP–PNP and Swi5–Sfr1.

## Discussion

In this study, we analyzed the Dmc1-driven DNA strand exchange reaction using real-time kinetic analysis systems. The results showed that the Dmc1-driven DNA strand exchange reaction proceeded in three steps involving two three-strand intermediates, namely C1 and C2 (Figures 1A–D). Abortive DNA pairing and displacement assays suggested that C1 consists of the donor dsDNA with its original base pairing and ssDNA in the presynaptic filament, while C2 consists of ssDNA in the presynaptic filament intertwined with the complementary strand of the donor dsDNA, leading to the formation of a new heteroduplex DNA (Figures 1E–J). These results demonstrate that the DNA strand exchange reaction driven by Dmc1 proceeds very similarly to the strand exchange reactions driven by Rad51 (28,30).

The strand exchange reactions with short dsDNA molecules (40 and 60 bp) were more strongly stimulated by AMP–PNP than by ATP, whereas in reactions initiated with long dsDNA (83 bp), the amount of final product was lower in the presence of AMP–PNP than in the presence of ATP (Figures 2A and B). Simulation analysis revealed that the *K*_1_ values with AMP–PNP were larger than those with ATP under all conditions. On the other hand, in reactions with AMP–PNP, the *K*_3_ and *K_total_* values were larger compared to those in reactions with ATP when short DNA was used. However, when long dsDNA was used, both values in the reactions containing either ATP or AMP–PNP were similarly small (Figures 2C). These results show that ATP hydrolysis is not essential for forming three-strand intermediates between homologous DNA, but is necessary for expanding heteroduplex DNA regions beyond a certain length. In a previous single-molecule analysis of the RecA-driven DNA strand exchange reaction, it was shown that the active synapsis region, where strand exchange occurs, is approximately 80 bp regardless of donor dsDNA length, and that strand exchange with donor dsDNA of several thousands bp is completed by ATP-hydrolysis-dependent migration of the region (36). It may be that the smallest unit in which strand exchange by fission yeast Dmc1 proceeds is about 80 bp, similar to that of RecA. Notably, fission yeast Rad51 failed to produce a final product when the dsDNA with 40 bp was used (28), suggesting that this minimum unit varies depending on the recombinase.

The analysis of the nucleotide dependency ssDNA binding by Dmc1 revealed that the *K_D_* values significantly differed in the presence of ATP, ADP, or AMP–PNP (Figures 6A and B). Real-time analysis of filament formation and dissociation showed a three-fold higher *V_N_* in the presence of AMP–PNP than in the presence of ATP, which strongly promoted filament nucleation (Figure 6E). Additionally, the Dmc1 filament with AMP–PNP was about 46 times more stable than that with ATP (Figure 6F). On the other hand, the ssDNA binding affinity of Rad51 was about four times stronger than that of Dmc1 in the presence of ATP and unaffected by other adenine nucleotides (Figures 6A, B, 7A and B). Interestingly, there was no difference in the stabilities of Dmc1 and Rad51 filaments in the presence of AMP–PNP (Figures 6F, 7G and H). These findings suggest that the turnover rate of Dmc1-ssDNA filament is faster than that of Rad51, depending on adenine nucleotide binding state. This idea is consistent with previous reports showing that human DMC1 maintains an ATP-bound active state on ssDNA, whereas human RAD51 rapidly accumulates the ADP-bound inactive form (23,25). Furthermore, this implies that regulating filament association is more crucial for Dmc1 than changing strand exchange activity after filament formation, unlike Rad51.

**Figure 8.**
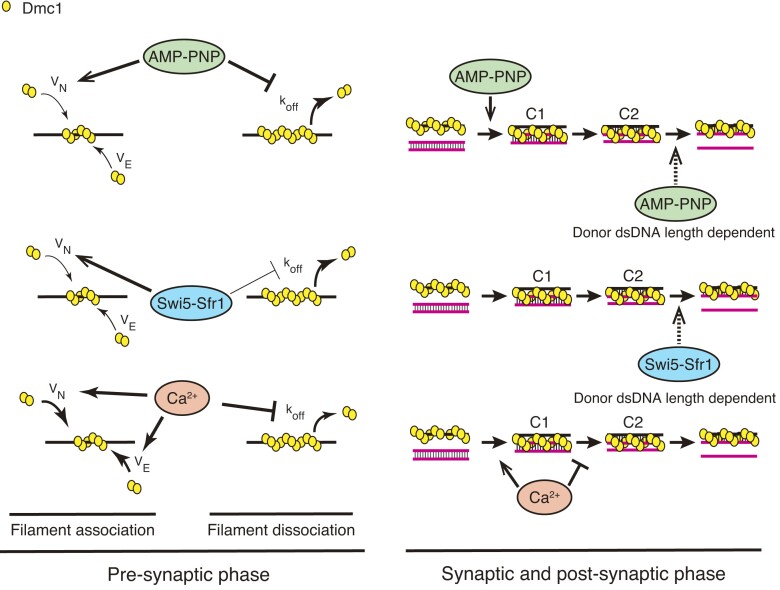
The distinct modes of action of Swi5–Sfr1, Ca^2+^ and AMP–PNP on Dmc1-driven DNA strand exchange. In the presynaptic phase, AMP–PNP stimulates filament nucleation and stabilizes the resultant filament. In the synaptic and postsynaptic phases, AMP–PNP stimulates the capture of donor dsDNA and facilitates the formation of the final product, but this occurs only with short donor dsDNA. Swi5–Sfr1 also promotes filament nucleation and stabilizes the filament, but this effect is much weaker than that of AMP–PNP. During the DNA strand exchange reaction, Swi5–Sfr1 helps to produce the final products when donor dsDNA is short. Ca^2+^ strongly enhances both filament nucleation and elongation and stabilizes the filament. The effects of Ca^2+^ surpass those of the two other factors. Although Ca^2+^ facilitates the C1 intermediate, it inhibits the subsequent reaction process.

The distinct effects of Swi5–Sfr1, AMP–PNP, and Ca^2+^ on Dmc1 and Rad51 filaments are depicted in Figure 8 and Supplementary Figure S5, respectively. Swi5–Sfr1 accelerated the C1–C2 transition and final product formation in the Rad51-driven DNA strand exchange reaction (Supplementary Figure S5) (28), while it stimulated product formation only at the final step and only with shorter donor dsDNA in the Dmc1-driven DNA strand exchange reaction. Pairing assays using various lengths of dsDNA demonstrated that stimulation of Rad51 strand exchange activity by Swi5–Sfr1 was stronger with longer donor dsDNA, unlike the strand exchange activity of Dmc1, which did not increase with the length of the donor DNA. Previous reports have indicated that Swi5–Sfr1 promotes ATP hydrolysis by Rad51 in a ssDNA-dependent manner but not ATP hydrolysis by Dmc1 (15,18,19). If the role of ATP hydrolysis by Rad51 and Dmc1 becomes more critical as the donor dsDNA length increases, the stimulation of ATPase activity by Swi5–Sfr1 may account for their distinct strand exchange activation mechanisms. In this regard, since the C-terminal domain of Sfr1 has been reported to be essential for ATPase activation in Rad51 (37), the interaction of this domain with Rad51 or Dmc1 is expected to be the key to elucidating the functions of Swi5–Sfr1 for the two recombinases.

In the presence of Swi5–Sfr1, the *K_D_* value of Dmc1 for ssDNA was reduced to a level similar to that observed with AMP–PNP (Figures 6A–D). Moreover, kinetic analysis of filament formation showed that Swi5–Sfr1 stimulated the filament nucleation phase (Figures 6E and 8). Interestingly, Swi5–Sfr1, at a concentration 1/10th of Dmc1, exhibited almost the same high-promoting effect as AMP–PNP. On the other hand, Swi5–Sfr1 stabilized Dmc1-ssDNA filaments, but this effect was much weaker than with AMP–PNP (Figure 6F). Although Swi5–Sfr1 inhibited Rad51 filament formation, it significantly stabilized Rad51-ssDNA filaments (Figures 7E–H and Supplementary Figure S5). A high concentration of Swi5–Sfr1 stabilized Rad51 filaments to a level almost equivalent to that in the presence of AMP–PNP. Previous biochemical analysis showed that fission yeast Swi5–Sfr1 increases the amount of Dmc1 protein bound to ssDNA and that Swi5–Sfr1 acts as a typical ‘mediator’ in the formation of Dmc1 filaments on RPA-coated ssDNA, but that it does not help Rad51 binding onto RPA-prebound ssDNA (15,18,19).

Given that the stabilization effect of Swi5–Sfr1 on Dmc1-ssDNA filaments is much weaker than on Rad51 filaments (Figures 6F, 7G and H), but Swi5–Sfr1 stimulates the association of Dmc1 on ssDNA (Figure 6E), and Dmc1 filament turnover is more likely to occur depending on the nucleotide-binding state of Dmc1, it is suggested that Swi5–Sfr1 maintains the filament in its active form by promoting Dmc1 rebinding. Swi5–Sfr1 may act as a molecular chaperone, similar to Rad55–Rad57 in the case of Rad51 as proposed by Roy et al (38), to maintain Dmc1 filaments, which dynamically turnover with ATP hydrolysis, in their active form rather than binding tightly to the filaments and acting as a stabilization factor. In light of this, both AMP–PNP and Swi5–Sfr1 would appear to maintain the active form of the Dmc1-ssDNA filament, but via very different mechanisms. When DNA strand pairing and displacement assays were conducted using various lengths of donor dsDNA in the presence of AMP–PNP or Swi5–Sfr1, the changes in *K_3_* and *K_tota_*_l_ values as dsDNA length increased were similar (Figures 2A, C, 4A and C), suggesting that the role of the two factors is to maintain the active form of the Dmc1 filament. On the other hand, C1 intermediate formation was particularly inhibited when AMP–PNP and Swi5–Sfr1 were added simultaneously. Based on biochemical and structural analysis, our group proposed that Swi5–Sfr1 binds to the Rad51 filament groove to stabilize it in its active form, but that excess amounts of Swi5–Sfr1 interfere with the interaction between the Rad51 filament and donor dsDNA during the homology search (37,39).

The binding of Swi5–Sfr1 to the stable Dmc1 filament in the presence of AMP–PNP may prevent the formation of the three-strand intermediate, as seen with the Rad51 filament (28). Notably, when AMP–PNP is present, Swi5–Sfr1 does not affect the pairing reaction driven by Rad51. In the presence of ATP, excess amounts of Swi5–Sfr1 inhibit dsDNA capture by Rad51 more strongly than by Dmc1 (Figure 3A–C) (15,19,28). These results suggest that the binding modes of Swi5–Sfr1 to Dmc1- and Rad51-ssDNA filaments are similar, but the activation mechanisms of these filaments are very different. Swi5–Sfr1 may stabilize the Rad51-ssDNA filament and regulate its activity by stimulating ATP hydrolysis by Rad51, while Swi5–Sfr1 maintains the Dmc1–ssDNA filament in its active form by promoting the association of Dmc1 on ssDNA.

The Hop2–Mnd1 complex, which exhibits structural similarities to Swi5–Sfr1, is another well-known accessory factor of Dmc1 in DNA strand exchange reactions (40–42). Hop2–Mnd1 promotes donor dsDNA capture by Dmc1, even between non-homologous sequences (40–42). Structural analysis revealed that the N-terminal regions of Hop2 and Mnd1 possess a dsDNA binding domain crucial for the stimulation of the dsDNA capturing activity of Dmc1, while the C-terminal region of Hop2 has an ssDNA binding domain that interacts with the Dmc1–ssDNA filament. Intriguingly, MD simulations showed that the dsDNA binding domains of Hop2 and Mnd1 distort and unfold the DNA double helix structure. Our recent biochemical reconstitution demonstrated that Swi5–Sfr1 and Hop2–Mnd1 synergistically stimulate Dmc1-driven strand exchange through different mechanisms, with Hop2–Mnd1 promoting the initiation of strand exchange (12,43). This step most likely corresponds to Step 1 defined in this study.

Ca^2+^ promoted the formation of the initial intermediate C1 but significantly inhibited the generation of heteroduplexes inside the C2 intermediate (Figures 5A, C and 8). Pairing assays using 2AP-labeled ssDNA also showed that in the presence of Ca^2+^, the formation of intermediates containing heteroduplexes was less efficient than in the presence of Mg^2+^ (Figures 5D–F). These results contrast with the fact that Ca^2+^ promoted the C1–C2 transition of the Rad51-driven reaction (28). Ca^2+^ significantly increased the affinity of Dmc1 for ssDNA, and the filament association rate in reactions with Ca^2+^ was much faster than in reactions with AMP–PNP or Swi5–Sfr1 (Figures 6A, B, E and 8).

Furthermore, in the filament dissociation assay, the filament was too stable to calculate the dissociation rate constant in the presence of Ca^2+^ (Figure 6F). In the case of Rad51, Ca^2+^ also promoted filament association, but the effect was weaker than for Dmc1 (Figures 6E, 7E, F and Supplementary Figure S5). Moreover, the stabilization of the Rad51 filament by Ca^2+^ was significantly weaker than that by AMP–PNP or Swi5–Sfr1 (Figures 7G and H). These results indicate that the effect of Ca^2+^ on Dmc1 cannot be explained simply by the inhibition of ATP hydrolysis activity of Dmc1 and that its effect on Dmc1 is entirely different from its effect on Rad51. After Dmc1–ssDNA filament capture of donor dsDNA in the presence of Ca^2+^, the filament may be unable to form a heteroduplex inside the intermediate due to the lack of flexibility in the filament.

A recent study employing the D-loop extension assay reported that the DNA polymerase could extend the D-loop region generated by Dmc1 only in the presence of Mg^2+^ and not in the presence of Ca^2+^ (44). By contrast, the DNA polymerase could extend the D-loop region generated by Rad51 in the presence of both Mg^2+^ and Ca^2+^. These results suggested that the reaction products generated by Dmc1, but not by Rad51, differ between reactions containing Mg^2+^ and Ca^2+^ (44). Further analysis of the C1 and C2 structures formed by Rad51 and Dmc1 in the presence of Mg^2+^ or Ca^2+^ should provide additional insights into the differences in the mechanisms of Rad51- and Dmc1-driven strand exchange.

Comparing the results of the three-step model analysis of DNA strand exchange reactions by Dmc1 and Rad51, the *K_2_* value of Dmc1 is larger than that of Rad51, indicating that the C1–C2 transition (step 2) is more likely to occur in Dmc1 than in Rad51. This property may favor Dmc1 for exchanging DNA strands between homologous chromosomes where mismatches can occur. On the other hand, the smaller *K_2_* value of Rad51 may be necessary for accurate recombination repair.

In fission yeast, meiotic DSB repair at strong hotspots predominantly occurs with the sister chromatid, resulting in few crossovers per DSB. This process requires Rad51 and Swi5–Sfr1 but not Dmc1. Conversely, at non-hotspot DSB sites, repair primarily occurs through interhomolog recombination, yielding more crossovers per DSB. This pathway necessitates Rad51, Dmc1, and Swi5–Sfr1. Therefore, Swi5–Sfr1 is essential for the functions of both Rad51 and Dmc1 in the meiotic recombination of fission yeast (45–49).

In sharp contrast, Mei5–Sae3, the budding yeast counterpart of Swi5–Sfr1, functions exclusively with Dmc1 (50,51). Notably, it has been proposed that the strand exchange activity of Rad51 is completely dispensable, with Rad51 acting solely as an accessory factor for Dmc1 in the meiotic recombination of budding yeast (52). Furthermore, it has been suggested that Mei5–Sae3 and Rad51 function independently in Dmc1 filament stability (53).

Taken together, the Dmc1 filament, whose formation is facilitated by Swi5–Sfr1, is the primary mechanism for DSB repair at non-hotspot sites to produce crossover recombinants in fission yeast. Meanwhile, at the hotspots, Swi5–Sfr1 stabilizes the Rad51 filament by preventing Rad51 dissociation, thus activating the filament. Swi5–Sfr1 associated with the Rad51 filament also facilitates the later stages of strand exchange, thereby enhancing the efficiency of DSB repair at the DSB hotspots. This proposed scenario is well-supported by both genetic and biochemical evidence obtained so far.

In conclusion, our work provides a comprehensive understanding of the molecular mechanism underlying Dmc1-driven DNA strand exchange, encompassing the initial Dmc1-ssDNA filament formation, subsequent dsDNA capture, and heteroduplex formation. Our findings indicate that each step is differently regulated by various factors, such as Swi5–Sfr1, ATP and Ca^2+^.

## Supplementary Material

gkae841_Supplemental_File

## Data Availability

The data of this study are available within the article and/or its supplementary data, or available upon reasonable request.

## Notes

Present address: Kentaro Ito, Graduate School of Medical Life Science, Yokohama City University, Yokohama, Kanagawa 230-0045, Japan.

Present address: Takahisa Maki, Faculty of Medical Science, University of Fukui, Fukui 910-1193, Japan.
