# Supplementary material for: The Swi5–Sfr1 complex regulates Dmc1- and Rad51-driven DNA strand exchange proceeding through two distinct three-stranded intermediates by different mechanisms
Source: Nucleic Acids Res. 2024 Sep 28;52(20):12517–33. doi: 10.1093/nar/gkae841 (PMC11551746; doi:10.1093/nar/gkae841)
Supplement: gkae841_Supplemental_File [file gkae841_supplemental_file.pdf]

The Swi5-Sfr1 complex regulates Dmc1- and Rad51-driven DNA strand exchange proceeding through two distinct three-stranded intermediates by different mechanisms.

by

Kentaro Ito, Takahisa Maki, Shuji Kanamaru, Masayuki Takahashi and Hiroshi Iwasaki

Supplementary Materials including

Supplementary Tables S1 – S7

Supplementary Figures S1- S5

Supplementary Table S1. Oligonucleotides used in this study

Name	Sequence (5' to 3')
16A(-)	AAATGAACATAAAGTAAATAAGTATAAGGATAATACAAAATAAGTAAAT GAATAAACATAGAAAATAAAGTAAAGGATATAAA
FAM-16A(-)	[FAM]AAATGAACATAAAGTAAATAAGTATAAGGATAATACAAAATAAGT AAATGAATAAACATAGAAAATAAAG TAAAGGATAT AAA
16A(+)[ROX]	TTTATATCCTTTACTTTATTTTCTATGTTTATTCATTTACTTATTTTGTA TTATCCTTATACTTATTTACTTTATGTTCATTT[ROX]
16A(−)_40 bp	AAATGAACATAAAGTAAATAAGTATAAGGATAATACAAAA
[FAM]16A(−)_40 bp	[FAM]AAATGAACATAAAGTAAATAAGTATAAGGATAATACAAAA
16A(+)[ROX]_40 bp	TTTTGTATTA TCCTTATACT TATTTACTTT ATGTTCATTT[ROX]
16A(−)_60 bp	AAA TGA ACA TAA AGT AAA TAA GTA TAA GGA TAA TAC AAA ATA AGT AAA TGA ATA AAC ATA
[FAM]16A(−)_60 bp	[FAM]AAA TGA ACA TAA AGT AAA TAA GTA TAA GGA TAA TAC AAA ATA AGT AAA TGA ATA AAC ATA
16A(+)[ROX]_60 bp	TATGTTTATT CATTTACTTA TTTTGTATTA TCCTTATACT TATTTACTTT ATGTTCATTT[ROX]
[TAMRA]dT72	[TAMRA]TTTTTTTTTTTTTTTTTTTTTTTTTTTTTTTTTTTTTTTTTTTTTT TTTTTTTTTTTTTTTTTTTTTTTTTT
16A(−)_3 × 2AP	AAATG[2AP]ACATAAAGTAAAT[2AP]AGTATAAGGATAAT[2AP]CAAAAT AAGTAAATGAATAAACATAGAAAATAAAGTAAAGGATATAAA

Supplementary Table S2. Maximum FRET efficiency, E_{max}

	DNA strand pairing		DNA strand displacement	
dsDNA (bp)	DNA only	Dmc1	DNA only	Dmc1
40	0.212 ± 0.005	0.251 ± 0.009	0.466 ± 0.015	0.449 ± 0.019
60	0.284 ± 0.057	0.293 ± 0.059	0.291 ± 0.017	0.320 ± 0.026
83	0.384 ± 0.023	0.390 ± 0.022	0.297 ± 0.016	0.298 ± 0.046

Each value is an average of the values of three independent experiments. ± indicates standard deviation.

Supplementary Table S3. Summary of parameters obtained from the experiments in Figure 2A

	k_1 × 10^5 (M^{-1} s^{-1})	k_{-1} × 10^{-2} (s^{-1})	K_1 × 10^6 (M^{-1})	k_2 × 10^{-3} (s^{-1})	k_{-2} × 10^{-3} (s^{-1})	K_2	k_3 × 10^{-3} (s^{-1})	k_{-3} × 10^5 (M^{-1} s^{-1})	K_3 × 10^{-8} (M)	K_{total} x 10^{-2}
40 bp dsDNA										
ATP	1.46±0.14	5.04±0.48	2.92±0.42	19.6±0.8	5.58±0.23	3.51±0.01	2.93±0.12	1.32±0.11	2.22±0.28	22.8±4.1
AMP-PNP	4.99±0.16	6.74±0.73	7.45±0.76	22.7±0.6	5.15±0.20	4.41±0.23	2.83±0.10	0.963±0.071	2.96±0.34	98.8±25.7
60 bp dsDNA										
ATP	1.35±0.32	3.41±0 .51	4.08±1.28	18.7±4.79	6.27±0.48	2.96±0.53	2.33±0.020	2.36±0.41	1.00±0.19	11.7±2.5
AMP-PNP	6.46±0.34	5.00±0.44	12.9±0.9	19.5±0.9	6.80±0.42	2.88±0.29	2.31±0.17	1.28±0.08	1.80±0.14	68.0±15.1
83 bp dsDNA										
ATP	19.2±2.9	12.7±1.0	15.2±3.5	13.5±2.8	10.1±1.0	1.31±0.15	2.61±0.07	8.76±1.84	0.306±0.064	6.14±1.93
AMP-PNP	28.7±0.9	11.4±0.5	3.52±0.20	4.25±0.24	4.75±0.53	4.78±0.98	1.05±0.28	5.94±0.77	0.183±0.074	4.07±1.39

Each value is the average of the values of three independent experiments. ± indicates standard deviation.

Supplementary Table S4. Summary of parameters obtained from the experiments in Figure 3A

[S5S1]/[Dmc1]	$k_1 \times 10^5$ (M^{-1} s^{-1})	$k_{-1} \times 10^{-2}$ (s^{-1})	$K_1 \times 10^6$ (M^{-1})	$k_2 \times 10^{-3}$ (s^{-1})	$k_{-2} \times 10^{-3}$ (s^{-1})	K_2	$k_3 \times 10^{-3}$ (s^{-1})	$k_{-3} \times 10^5$ (M^{-1} s^{-1})	$K_3 \times 10^{-8}$ (M)	K_{total} x 10^{-2}
0	1.35±0.32	3.41±0 .51	4.08±1.28	18.7±4.79	6.27±0.48	2.96±0.53	2.33±0.020	2.36±0.41	1.00±0.19	11.7±2.5
0.02	2.45±0.43	4.31±0 .45	5.78±1.56	21.9±4.7	5.25±0.98	4.16±0.17	3.14±0.36	1.72±0.42	1.88±0.44	44.3±10.0
0.05	2.62±0.41	4.74±0 .14	5.51±0.73	23.3±0.6	5.76±0.53	4.04±0.27	3.38±0.19	1.75±0.53	2.04±0.54	47.2±18.8
0.1	2.47±0.57	4.63±0 .26	5.37±1.39	20.3±1.8	5.11±0.31	3.98±0.48	3.46±0.04	1.13±0.12	3.07±0.34	64.7±14.2
0.2	1.75±0.20	3.93±0 .91	4.63±1.22	16.7±2.9	5.08±0.54	3.27±0.22	3.39±0.43	0.994±0.086	3.43±0.57	50.9±10.4
0.5	2.31±0.52	6.36±1.74	3.66±0.21	15.1±0.8	6.27±0.83	2.51±0.34	3.51±0.15	0.879±0.067	4.01±0.42	36.7±4.0

Each value is the average of the values of three independent experiments. ± indicates standard deviation. S5S1, Swi5-Sfr1.

Supplementary Table S5. Summary of parameters obtained from the experiments in Figure 3D

	$k_1 \times 10^5$ ($M^{-1}\ s^{-1}$)	$k_{-1} \times 10^{-2}$ (s^{-1})	K_1 x 10^6 (M^{-1})	$k_2 \times 10^{-3}$ (s^{-1})	$k_{-2} \times 10^{-3}$ (s^{-1})	K_2	$k_3 \times 10^{-3}$ (s^{-1})	$k_{-3} \times 10^5$ ($M^{-1}\ s^{-1}$)	K_3 x 10^{-8} (M)	K_{total} x 10^{-2}
ATP	1.35±0.32	3.41±0 .51	4.08±1.28	18.7±4.79	6.27±0.48	2.96±0.53	2.33±0.020	2.36±0.41	1.00±0.19	11.7±2.5
AMP-PNP	6.46±0.34	5.00±0.44	12.9±0.9	19.5±0.9	6.80±0.42	2.88±0.29	2.31±0.17	1.28±0.08	1.80±0.14	68.0±15.1
ATP +S5S1	2.47±0.57	4.63±0 .26	5.37±1.39	20.3±1.8	5.11±0.31	3.98±0.48	3.46±0.04	1.13±0.12	3.07±0.34	64.7±14.2
AMP-PNP +S5S1	1.12±0.12	3.82±0 .25	2.95±0.52	13.3±2.0	4.72±0.43	2.80±0.18	2.24±0.37	1.65±0.12	1.35±0.16	11.0±1.4

Each value is the average of the values of three experiments. ± indicates standard deviation. S5S1, Swi5-Sfr1.

Supplementary Table S6. Summary of parameters obtained from the experiments in Figure 4A

	$k_1 \times 10^5$ (M^{-1} s^{-1})	$k_{-1} \times 10^{-2}$ (s^{-1})	$K_1 \times 10^6$ (M^{-1})	$k_2 \times 10^{-3}$ (s^{-1})	$k_{-2} \times 10^{-3}$ (s^{-1})	K_2	$k_3 \times 10^{-3}$ (s^{-1})	$k_{-3} \times 10^5$ (M^{-1} s^{-1})	$K_3 \times 10^{-8}$ (M)	K_{total} x 10^{-2}
40 bp dsDNA										
-S5S1	1.46±0.14	5.04±0.48	2.92±0.42	19.6±0.8	5.58±0.23	3.51±0.01	2.93±0.12	1.32±0.11	2.22±0.28	22.8±4.1
+S5S1	2.08±0.39	4.59±0.20	4.52±0.68	19.4±0.9	4.47±0.38	4.37±0.48	3.40±0.36	0.926±0.125	3.74±0.85	72.1±5.0
83 bp dsDNA										
-S5S1	19.2±2.9	12.7±1.0	15.2±3.5	13.5±2.8	10.1±1.0	1.31±0.15	2.61±0.07	8.76±1.84	0.306±0.064	6.14±1.93
+S5S1	19.7±1.0	10.6±0.2	18.6±1.3	9.39±0.26	7.10±0.18	1.32±0.03	2.30±0.11	4.84±0.41	0.476±0.017	11.7±1.2

Each value is the average of the values of three experiments. ± indicates standard deviation. S5S1, Swi5-Sfr1.

Supplementary Table S7. Summary of parameters obtained from the experiments in Figure 5A

	$k_1 \times 10^5$ (M^{-1} s^{-1})	$k_{-1} \times 10^{-2}$ (s^{-1})	$K_1 \times 10^6$ (M^{-1})	$k_2 \times 10^{-3}$ (s^{-1})	$k_{-2} \times 10^{-3}$ (s^{-1})	K_2	$k_3 \times 10^{-3}$ (s^{-1})	$k_{-3} \times 10^5$ (M^{-1} s^{-1})	$K_3 \times 10^{-8}$ (M)	K_{total} x 10^{-2}
40 bp dsDNA										
Ca^{2+}	8.38±0.59	5.78±0.40	14.5±1.8	13.4±2.3	20.3±1.1	0.658±0.08	0.819±0.08	3.85±0.62	0.218±0.05	2.10±0.71
60 bp dsDNA										
Ca^{2+}	11.1±0.4	4.15±0.09	26.8±0.8	8.02±0.71	13.0±0.3	0.614±0.05	1.17±0.05	3.15±0.47	0.376±0.05	6.20±1.13
83 bp dsDNA										
Ca^{2+}	16.8±0.5	8.15±0.73	20.7±1.7	6.82±0.33	10.2±1.4	0.673±0.06	1.42±0.15	3.36±0.38	0.428±0.06	5.94±0.73

Each value is the average of the values of three experiments. ± indicates standard deviation.

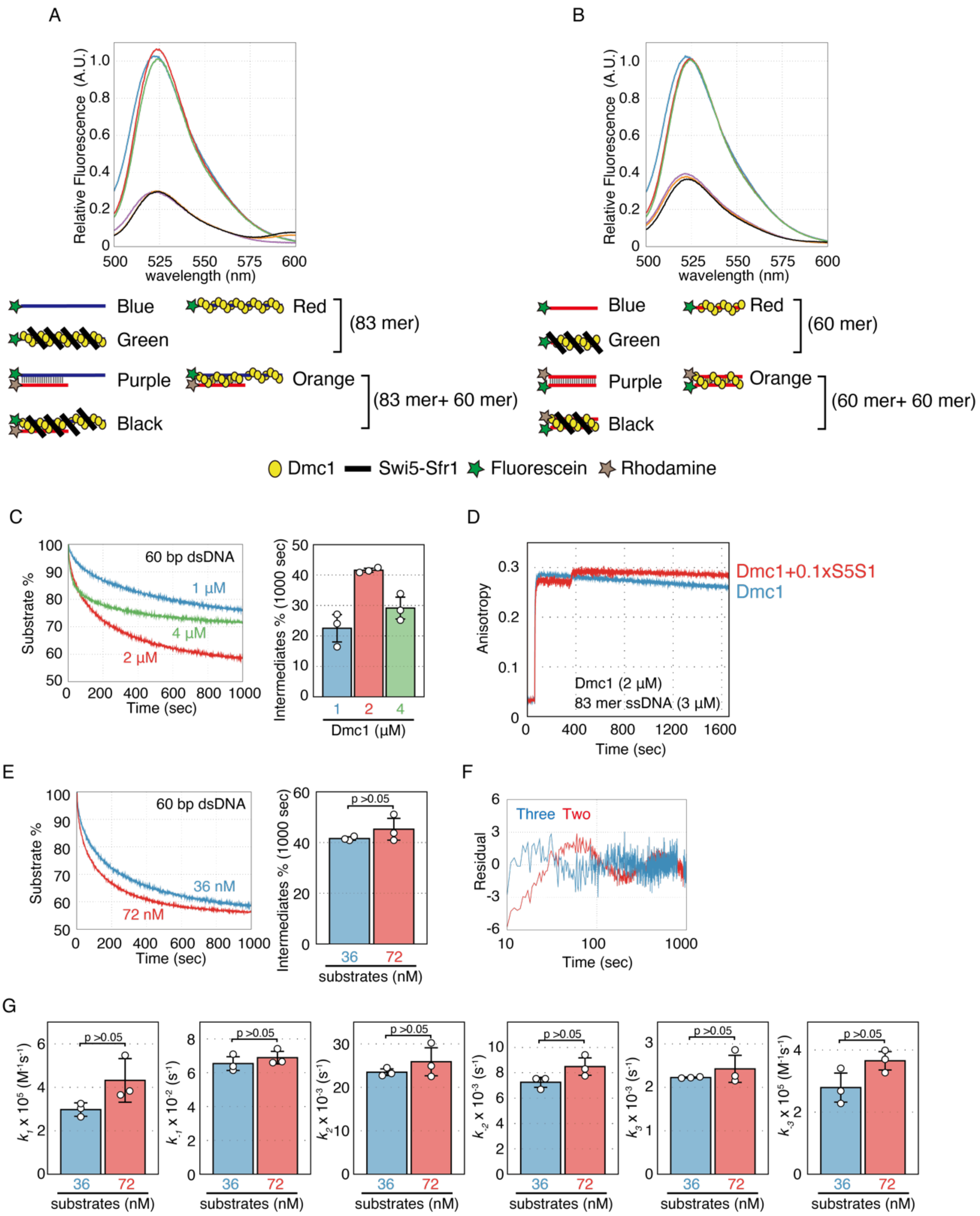

Supplementary Figure S1. Dmc1 does not influence the fluorescein emission or FRET between fluorescein and rhodamine.

Fluorescence spectra of substrates and final products of DNA strand pairing assays. **A** DNA strand pairing assays. Blue: 83-mer naked fluorescein-labeled ssDNA. Red: 83-mer fluorescein-labeled ssDNA with Dmc1. Green: 83-mer fluorescein-labeled ssDNA with Dmc1 and Swi5-Sfr1. Purple: Naked dsDNA containing 83-mer fluorescein-labeled ssDNA and 60-mer rhodamine-labeled

ssDNA. Orange: dsDNA containing 83-mer fluorescein-labeled ssDNA and 60-mer rhodamine-labeled ssDNA with Dmc1. Black, dsDNA containing 83-mer fluorescein-labeled ssDNA and 60-mer rhodamine-labeled ssDNA with Dmc1and Swi5-Sfr1. **B** DNA strand dissociation assays. Blue: 60-mer naked fluorescein-labeled ssDNA. Red: 60-mer fluorescein-labeled ssDNA with Dmc1, Green: 60-mer fluorescein-labeled ssDNA with Dmc1 and Swi5-Sfr1, Purple: naked dsDNA containing 60-mer fluorescein-labeled ssDNA and 60-mer rhodamine-labeled ssDNA, Orange: dsDNA containing 60-mer fluorescein-labeled ssDNA and 60-mer rhodamine-labeled ssDNA with Dmc1, Black, dsDNA containing 60-mer fluorescein-labeled ssDNA and 60-mer rhodamine-labeled ssDNA with Dmc1 and Swi5-Sfr1. **C** Left panel; time course of DNA strand pairing reactions containing 1 (blue), 2 (red) or 4 μM (green) of Dmc1. The conditions of these experiments were the same as the standard conditions except for the Dmc1 concentration. Right panel; Comparison of the intermediates generated in the pairing reaction containing 1 μM (blue), 2 μM (red) or 4 μM (green) of Dmc1. Intermediates % were obtained from the product % at 1000 sec after the initiation of the reaction. **D** The filament stability under pairing and displacement assays was tested by measuring the fluorescence anisotropy of fluorescein for 1600 sec, demonstrating that the Dmc1-ssDNA filaments were stable during these assays. **E** Left panel; time course of DNA strand pairing reactions containing 36 (blue) or 72 nM (red) of the substrates. The conditions of these experiments were the same as the standard conditions except for the substrates concentrations. Right panel; Comparison of the intermediates generated in the pairing reaction containing 36 (blue) or 72 nM (red) of the substrates. Intermediates % were obtained from the product % at 1000 sec after the initiation of the reaction. **F** Residuals between experimental data and a theoretical curve simulated using DynaFit for the pairing assay containing 72 nM of the substrates shown in **Supplementary Figure S1E**. Blue: residuals between the experimental data and the three-step model. Red: residuals between the experimental data and the two-step model. **G** The reaction rate constants of each reaction in **Supplementary Figure S1E** were calculated by simulation using the three-step model. Blue: reactions containing 36 nM of the substrates. Red: reactions containing 72 nM of the substrates. Data shown in **Supplementary Figure S1C**, **E** and **G** are average values ± s.d. (n = 3 independent experiments). Statistical analysis was performed using a two-tailed Student's t-test.

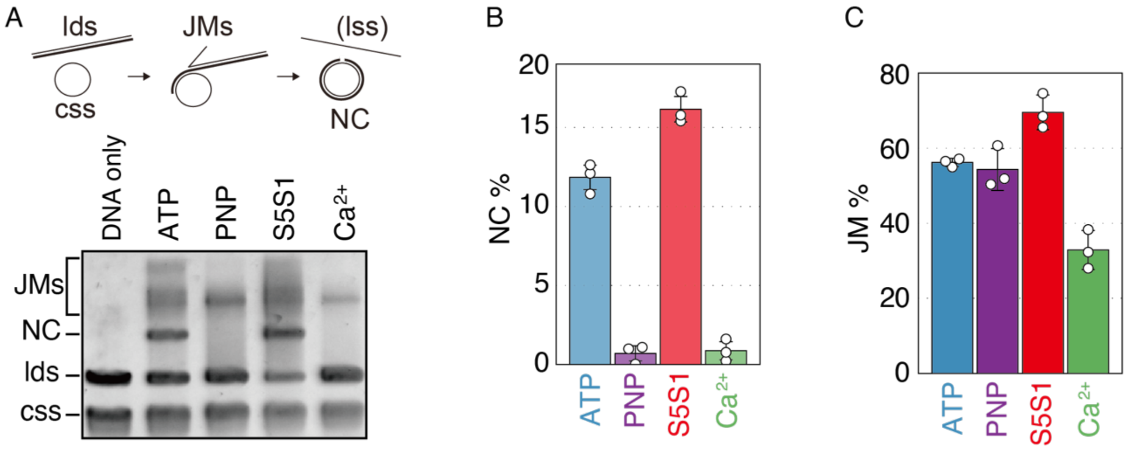

Supplementary Figure S2. Three-strand exchange assay using plasmid-sized DNA substrates from *φ*X174. A Upper: Schematics of the reaction. Pairing of circular single-stranded DNA from viral virion (css) with homologous linear double-stranded DNA from replicative form DNA (lds) generates joint molecules (JMs) as reaction intermediates. Subsequently, JM is converted to nicked circular DNA (NC) and linear ssDNA (lss) products. Note that lss is not, or is hardly, detectable by agarose gel electrophoresis. Lower: An agarose gel image of the three-strand exchange assay. First, Dmc1 (15 µM) and cssDNA (30 µM, nucleotide concentration) were mixed in buffer C (30 mM Tris-HCl [pH 7.5], 3 mM divalent ion ($MgCl_2$ or $CaCl_2$), 1 mM DTT, and 5% glycerol) containing 150 mM KCl, 1 mM adenine nucleotide (ATP or AMP-PNP) (Sigma) and an ATP regeneration system (8 mM creatine phosphate and 8 U/ml creatine kinase). The mixture was incubated for 5 min at 30°C. Subsequently, 0.15 µM Swi5-Sfr1 was added, and the mixture was incubated for another 5 min. Then, 1 µM RPA was added, followed by an additional 5-min incubation. After this, 10 µM (nucleotide concentration) ldsDNA linearized by ApaLI was added and the mixture was incubated for 2 h at 30°C. Psoralen (200 µg) was then added to the mixture, which was exposed UV to cross-link and fix DNA species, including reaction intermediates and final products. Following DNA cross-linking, a reaction stop solution containing 5.3% SDS and 6.6 mg/ml proteinase K (Takara) was added, and the mixture was incubated for 1 h at 30 °C. After deproteinization, the mixture was analyzed by 1% agarose gel electrophoresis, and the gel was stained by SYBR-Gold (Thermo Fisher Scientific). The reactions containing ATP, AMP-PNP, and Swi5-Sfr1, all of which included Mg^{2+}, were displayed as ATP, PNP, and S5S1, respectively, while the reaction containing ATP and Ca^{2+} (instead of ATP and Mg^{2+}) was displayed as Ca^{2+}. **B** Quantified yield of NC, reaction intermediates, form **Supplementary Figure S2A**. Blue: reaction containing ATP. Purple: PNP. Red: S5S1. Green: Ca^{2+}. **C** Quantified yield of JM, one of the final products, form **Supplementary Figure S2A**. Blue: reaction containing ATP. Purple: PNP. Red: S5S1. Green: Ca^{2+}. Data shown in **Supplementary Figures S2B** and **C** are average values ± s.d. (n = 3 independent experiments).

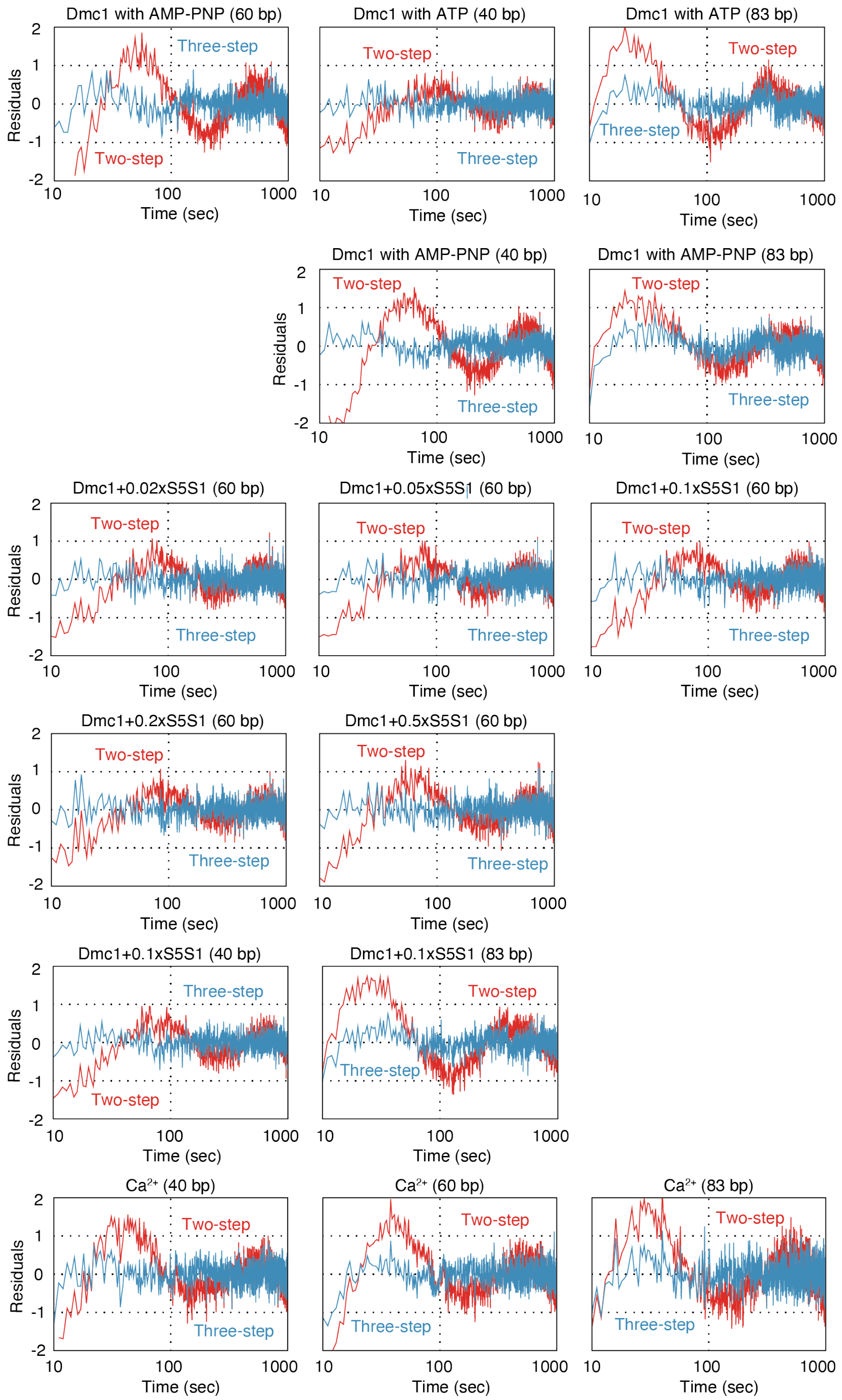

Supplementary Figure S3. DNA strand exchange fits a three-step reaction model. Residuals between experimental data from DNA strand pairing assays and theoretical curves from simulations of Three-Step (Blue) or Two-Step (Red) reactions using DynaFit.

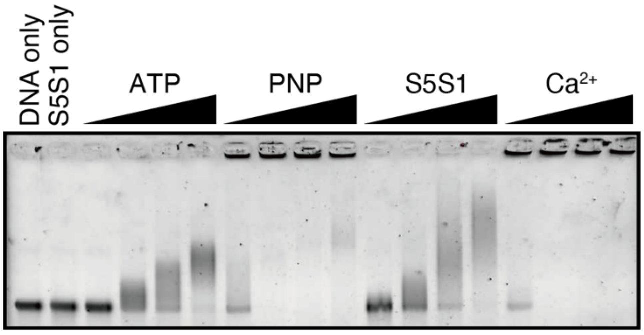

Supplementary Figure S4. EMSA for Dmc1. Various concentrations of Dmc1 (1.25, 2.5, 5 or 7.5 μM) were incubated with circular single-stranded DNA from φX174 (10 μM, nucleotide concentration) in buffer B (30 mM HEPES-KOH [pH 7.5], 100 mM KCl, 3 mM $MgCl_2$, 1 mM DTT, and 5% glycerol) containing 1 mM adenine nucleotide (ATP or AMP-PNP) for 5 min at 30°C. Swi5-Sfr1 (0.15 μM) was added after ATP addition when indicated. The mixtures were further incubated for 10 min at 30°C. After the 10 min incubation, glutaraldehyde (0.2% [w/w]) was added to the mixture. The mixture was incubated for 5 min and analyzed by a 1% agarose gel electrophoresis. The gel was stained with SYBR-Gold.

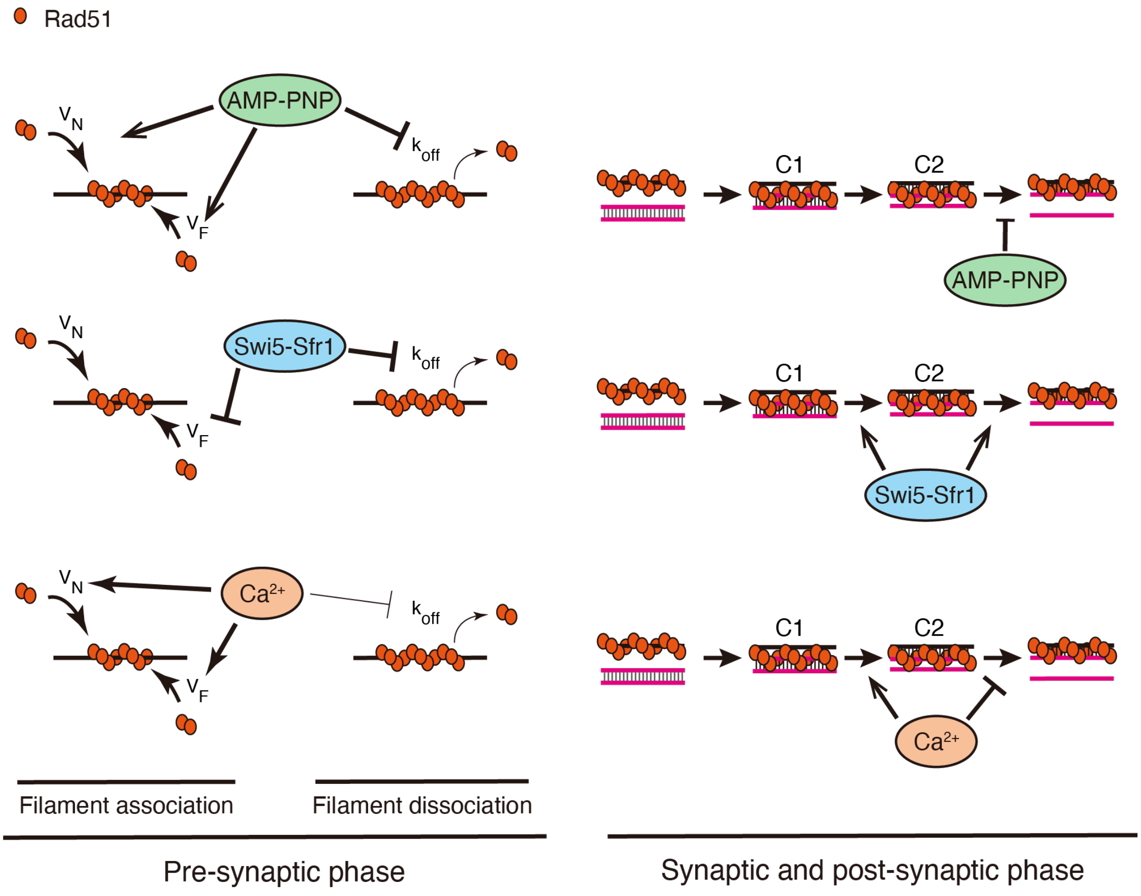

Supplementary Figure S5. The distinct modes of action of Swi5-Sfr1, Ca^{2+}, and AMP-PNP on Rad51-driven DNA strand exchange.

In the presynaptic phase, AMP-PNP facilitates filament nucleation and elongation by Rad51 on ssDNA and stabilizes the filament. However, in the presence of AMP-PNP, Rad51 cannot complete the DNA strand exchange reaction even with 40 bp dsDNA. Despite inhibiting filament formation of Rad51 on ssDNA, particularly during filament elongation, Swi5-Sfr1 stabilizes the filament. In the DNA strand exchange reaction, Swi5-Sfr1 promotes the C1-C2 transition and the formation of the final products. Ca^{2+} stimulates filament nucleation and elongation by Rad51 on ssDNA in a manner similar to AMP-PNP, but its stabilization effect on Rad51 is weaker. While Ca^{2+} enhances the C1-C2 transition, it inhibits the final product formation.
